# BBB-Crossing Ionizable Upconversion Nanoparticles for Synergistic Therapy of Carbapenem-Resistant Central Nervous System Infection

**DOI:** 10.34133/research.0946

**Published:** 2025-11-24

**Authors:** Xikang Tang, Yan Zhong, Xiaoli Yang, Yili Chen, Yawen Song, Quanshi Lin, Yougang Mai, Wojciech Chrzanowski, Zhijin Fan, Yuhui Liao

**Affiliations:** ^1^ Department of Pediatrics, Sun Yat-sen Memorial Hospital, Sun Yat-sen University, Guangzhou 510120, China.; ^2^ Institute for Engineering Medicine, Kunming Medical University, Kunming 650500, China.; ^3^ Department of Respiratory and Critical Care Medicine, Second Affiliated Hospital of Third Military Medical University (Army Medical University), Chongqing 400037, China.; ^4^ Department of Laboratory Medicine, The First Affiliated Hospital of Sun Yat-sen University, Guangzhou 510062, China.; ^5^ School of Materials Science and Engineering, Nanyang Technological University, Nanyang 639798, Singapore.; ^6^ Department of Clinical Laboratory, The Third Affiliated Hospital of Guangzhou Medical University, Guangzhou 510150, China.; ^7^ Sydney Pharmacy School, Faculty of Medicine and Health, The University of Sydney, Camperdown, NSW 2006, Australia.; ^8^ Division of Biomedical Engineering, Department of Materials Science and Engineering, Uppsala University, 75105 Uppsala, Sweden.

## Abstract

Central nervous system (CNS) infections caused by carbapenem-resistant Enterobacteriaceae (CRE) biofilms pose a major critical healthcare challenge. These infections are difficult to treat due to the rising prevalence of antibiotic resistance and the restrictive nature of the blood–brain barrier (BBB), which limits therapeutic access to the brain tissue. To address this, we developed an innovative pH-responsive nanotherapeutic platform, UC@MOF@RB+MEM, integrating upconversion nanoparticles (UCNPs), a metal–organic framework (MOF), the photosensitizer rose bengal (RB), and the antibiotic meropenem (MEM). This design exhibited dual responsiveness to both pH and near-infrared (NIR) light, creating synergistic effects that disrupted biofilm and eradicated bacteria while mitigating neuroinflammation. During CNS inflammation, BBB permeability increased, enabling the ionizable UC@MOF@RB+MEM nanoparticles to cross the BBB and target infection sites. In the acidic microenvironment of the infection, these nanoparticles released MEM, RB, and Zn^2+^. Under 980-nm NIR light irradiation, RB generated reactive oxygen species (ROS), effectively disrupting the biofilms and bacterial membranes while inhibiting carbapenemase activity. The synergistic effect of photodynamic therapy (PDT) combined with MEM enabled rapid and effective eradication of CRE biofilm-associated bacteria. Simultaneously, the released Zn^2+^ mitigated nerve cell damage through anti-inflammatory and antioxidant effects. The dual actions markedly enhanced the efficacy of UC@MOF@RB+MEM in treating CNS infections in mouse models, offering promising prospects for managing CNS infections caused by CRE strains, and extending its potential to other hard-to-treat, antimicrobial-resistant infections.

## Introduction

Central nervous system (CNS) infections present a significant clinical challenge due to the impermeability of the blood–brain barrier (BBB) to many antibacterial drugs [1,2]. Meropenem (MEM), owing to its broad-spectrum activity and high BBB penetration, serves as a primary therapeutic option for managing CNS infections in clinical practice [3,4]. However, the emergence of carbapenem-resistant organisms (CROs) in recent years has restricted the utilization of this medication, leading to poor prognosis and increased mortality rates [5–7]. Global epidemiological data indicate that among Gram-negative bacteria, the annual death toll attributable to carbapenem resistance increased by 89,200 from 1990 to 2021, exceeding that of any other antibiotic category during the same period [8]. The World Health Organization (WHO) has classified carbapenem-resistant Enterobacteriaceae (CRE) as a critical priority pathogen, highlighting the urgent need for the development of novel antibacterial drugs [9,10].

The main challenge in treating CRE infections is the formation of biofilms, which shield bacteria from the host immune system and reduce the efficacy of antibiotic interventions [11]. The dense structure of biofilms (composed of polysaccharides, proteins, and nucleic acids) poses a major barrier to treatment, limiting antibiotic penetration and enabling persistent bacterial survival. Bacteria within biofilms can also develop resistance, making eradication especially difficult. Drug resistance in biofilm-associated bacteria exhibits 10- to 1,000-fold increases compared to planktonic bacteria [12]. Effective therapies must therefore disrupt the biofilm matrix and eliminate the embedded bacteria.

Nanocarriers have demonstrated high efficacy in targeted drug delivery. In recent years, nanotechnology has been widely applied to research on cancer treatment and the management of drug-resistant bacterial infections [13–17]. Photodynamic therapy (PDT), a noninvasive modality with negligible systemic toxicity and low risk of drug resistance [18–20], utilizes light-activated photosensitizers (PSs) to generate reactive oxygen species (ROS) through oxygen sensitization [21–23]. Notably, ROS can potentiate the bactericidal efficacy of antibiotics against multidrug-resistant pathogens [24]. Integrating these advantages, we propose a spatiotemporally regulated nanosystem for co-delivering PSs and antibiotics, aiming to synergize ROS-enhanced antibacterial effects with stimuli-responsive drug release against CRE biofilm-associated CNS infections.

The microenvironment of CNS infections differs significantly from that of normal tissue, exhibiting distinctive physicochemical properties [25,26]. A notable feature of this altered environment is the development of an acidic microenvironment resulting from the abnormal accumulation of substances such as lactic acid, pyruvic acid, and carbon dioxide. Currently, pH-responsive nanoparticles have been developed as a highly promising therapeutic strategy [27–29]. These nanoparticles were modified with pH-sensitive groups that enable targeted drug release within the acidic microenvironment, thereby improving both the efficiency and efficacy of treatment [30]. This innovative drug release mechanism holds potential for application in treating various types of infections, including those affecting the CNS.

**Fig. 1.**
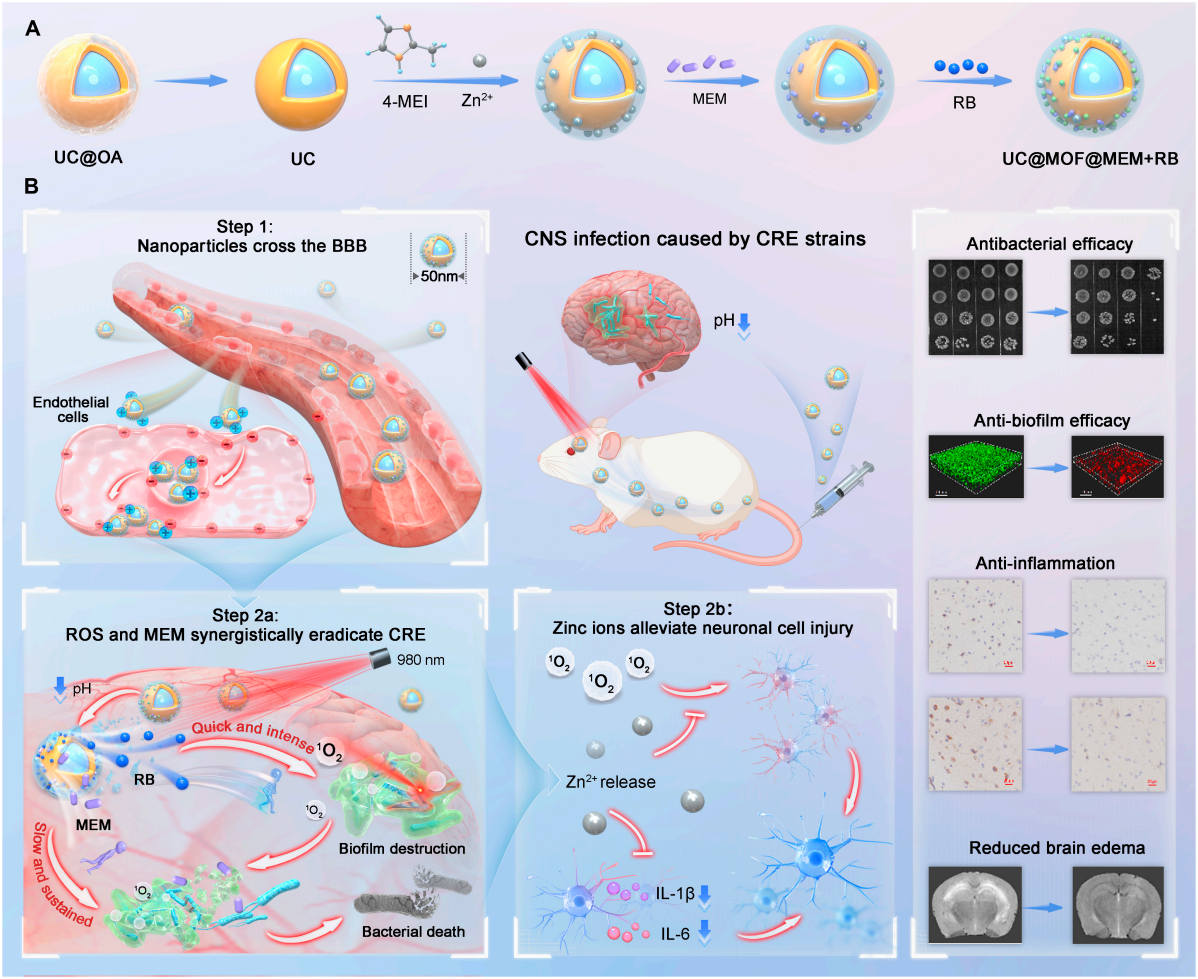
Schematic diagram illustrating the mechanism of UC@MOF@RB+MEM in treating CNS infections. (A) Structural fabrication: UCNP core synthesis, ZIF-8 coating formation through Zn-imidazole coordination, and co-loading of RB with MEM. (B) Therapeutic workflow: Step 1—BBB penetration enhanced by microenvironment-triggered protonation; Step 2a—biofilm structural disruption with planktonic bacterial eradication via PDT-MEM synergy; Step 2b—Zn^2+^-mediated neural protection through oxidative stress reduction and anti-inflammatory signaling.

Herein, we developed a pH-responsive photodynamic nanosystem combined with antibiotic treatment, as an innovative therapeutic strategy for CNS infections (Fig. 1). The nanodrug UC@MOF@RB+MEM, with upconversion nanoparticles (UCNPs) as the core, is coated with porous ZIF-8 coating, which can adsorb the PS RB and MEM. The ZIF-8 coating is formed by the mineralization of zinc acetate and 2-methylimidazole, which protonates the nanoparticles with a positive charge under acidic conditions, thereby enhancing the capacity to penetrate the BBB (Step 1). The 2-methylimidazole-mediated pH response also controls the optimal release of RB, MEM, and Zn^2+^ in the infected CNS. Upon 980-nm near-infrared (NIR) light irradiation, UCNPs activate RB to generate singlet oxygen for PDT. We showed that PDT disrupted the structural integrity of biofilms and bacterial membranes, and reduced carbapenemase activity, enabling more MEM to enter the cells without hydrolysis, thereby facilitating rapid bacterial eradication (Step 2a). Simultaneously, the released Zn^2+^ reduced inflammation and oxidative stress, hence preventing neuronal cell damage (Step 2b), ultimately achieving effective treatment outcomes for CNS infections. This study proposes an innovative strategy to overcome the MEM resistance, providing a promising solution for CNS infection management in the post-antibiotic era.

## Results and Discussion

### Preparation and characterization of UC@MOF@RB+MEM

**Fig. 2.**
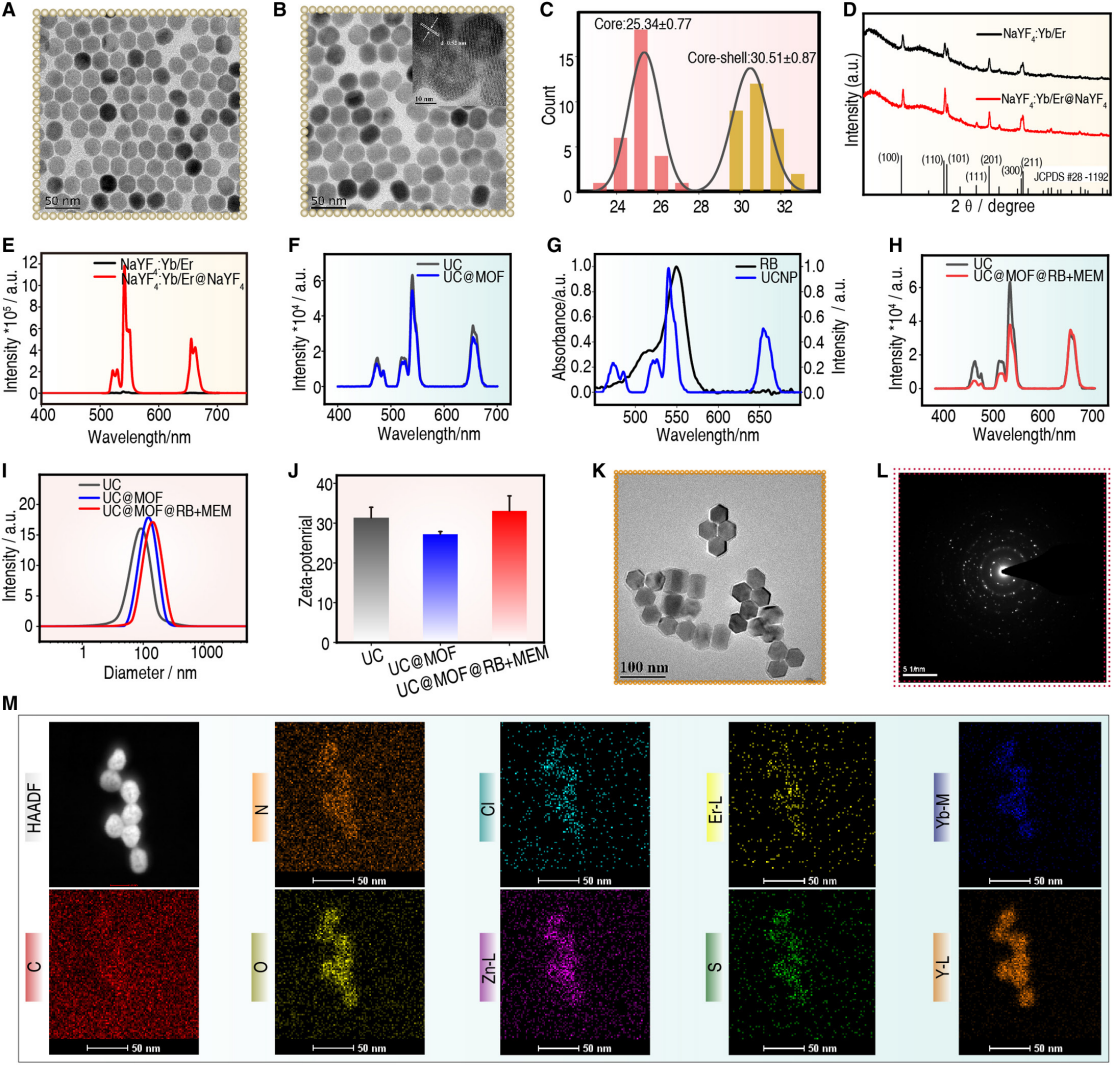
Characterization of nanomaterials. (A and B) TEM images and (C) particle size of core and core–shell UCNPs. The inset picture in (B) shows the high-resolution TEM image of UCNPs. (D) XRD pattern and (E) UCL spectra of core and core–shell UCNPs. (F) UCL spectrum of UC and UC@MOF. (G) UCL spectrum of the UCNPs under 980-nm excitation and UV–visible absorption spectrum of RB. (H) UCL spectrum of UC and UC@MOF@RB+MEM. (I) DLS analysis and (J) zeta potential value of UC, UC@MOF, and UC@MOF@RB+MEM. (K) TEM image. (L) Selected-area electron diffraction pattern. (M) High-angle annular dark-field scanning transmission electron microscopy (HAADF-STEM) image and corresponding elemental mapping images.

To shift the PDT activation window, core–shell structured UCNPs (NaYF_4_: Yb/Er@NaYF_4_) were synthesized via a thermal decomposition method. Er^3+^ and Yb^3+^ ions were co-doped into the core to generate green emission under 980-nm NIR light excitation, while an outermost layer of NaYF_4_ shell was grown to minimize the surface quenching effect. Transmission electron microscopy (TEM) images showed uniform dispersion and narrow size distribution of core and core–shell structured UCNPs (Fig. 2A and B), with an average diameter of 25 and 30 nm, respectively (Fig. 2C). Nanoparticles below 100 nm are considered to be more capable of crossing the BBB [31–33]. High-resolution TEM image showed a lattice spacing of 0.52 nm identified as the (100) crystal plane of the hexagonal phase (Fig. 2B, inset). Further confirmation of the crystal structure of UCNPs was provided by x-ray diffraction (XRD) analysis. All diffraction peaks in Fig. 2D can be indexed to the hexagonal phased UCNPs (JCPDS no. 28-1192).

Subsequently, the upconversion properties of the nanoparticles were verified. Figure 2E shows the up-conversion luminescence (UCL) spectra of UCNPs after irradiation with NIR light. Under 980-nm NIR light irradiation, the dominant spectrum exhibited visible green emission (525 and 541 nm) corresponding to the ^2^H_11/2_→^4^I_15/2_ and ^4^S_3/2_→^4^I_15/2_ transitions of Er^3+^, and red emission (656 nm) corresponding to the ^4^F_9/2_→^4^I_15/2_ transition of Er^3+^. In addition, the surface loading of MOF had no obvious effect on the UCL intensity of UC@MOF (Fig. 2F). Due to the overlap between the green UCL band of UCNPs and the absorption spectra RB (Fig. 2G), upon further loading of RB onto the surface of UCNP@MOF, the green UCL of UC@MOF@RB+MEM was significantly quenched, while the red UCL was hardly affected (Fig. 2H), demonstrating the feasibility of NIR light-activated PDT.

The dynamic light scattering (DLS) results demonstrated a gradual increase in particle size with each assembly step, confirming the successful construction of the nanoparticles (Fig. 2I). The coating of ZIF-8 was meant to reduce the surface potential of UCNP, which was confirmed by our experiments (Fig. 2J). The coated nanoparticles retain a positive charge, likely due to carbon dioxide dissolution in ultra-pure water, causing slight acidity that protonates ZIF-8. In blood, this weak positive charge attracts negatively charged proteins, forming a negatively charged surface/corona (Fig. S1A).

To better simulate physiological conditions, fetal bovine serum (FBS) was used, and the nanoparticles exhibited a hydrodynamic size of 87.9 nm with a negative surface charge (Fig. S1B and C). This negative charge is beneficial for prolonged circulation in the bloodstream. Upon reaching the infection site, the acidic microenvironment causes the imidazole groups on the ZIF-8 shell to deprotonate, increasing the nanoparticle’s positive charge (Fig. S1D). Notably, positively charged nanoparticles have been reported to facilitate transport across the BBB [34], suggesting that this charge reversal at the infection site may enhance their ability to penetrate the BBB and deliver drugs more effectively to the brain in vivo. It is worth noting that positive charges are relatively unstable and tend to interact with blood proteins to form a protein corona, which may hinder nanoparticle penetration across the BBB. In addition, such charge alterations could potentially influence the systemic toxicity of the nanoparticles. We anticipate that future studies will provide further clarification of these effects.

To further validate the ZIF-8 coating on the UCNP surface, TEM analysis was performed. TEM images of UC@MOF@RB+MEM showed a distinct shell structure surrounding the UCNP core, confirming the successful formation of the ZIF-8 coating (Fig. 2K). Electron diffraction patterns collected from different areas also confirmed the crystal structure of UCNP (Fig. 2L), matching the XRD data. Element mapping (Fig. 2M) and energy-dispersive x-ray spectroscopy (EDX) (Fig. S1E) identified the presence of encapsulating materials, including ZIF-8 (characterized by Zn), RB (indicated by Cl), and MEM (indicated by S), providing evidence of the successful assembly of UC@MOF@RB+MEM.

### In vitro antibacterial and anti-biofilm efficacy of UC@MOF@RB+MEM

**Fig. 3.**
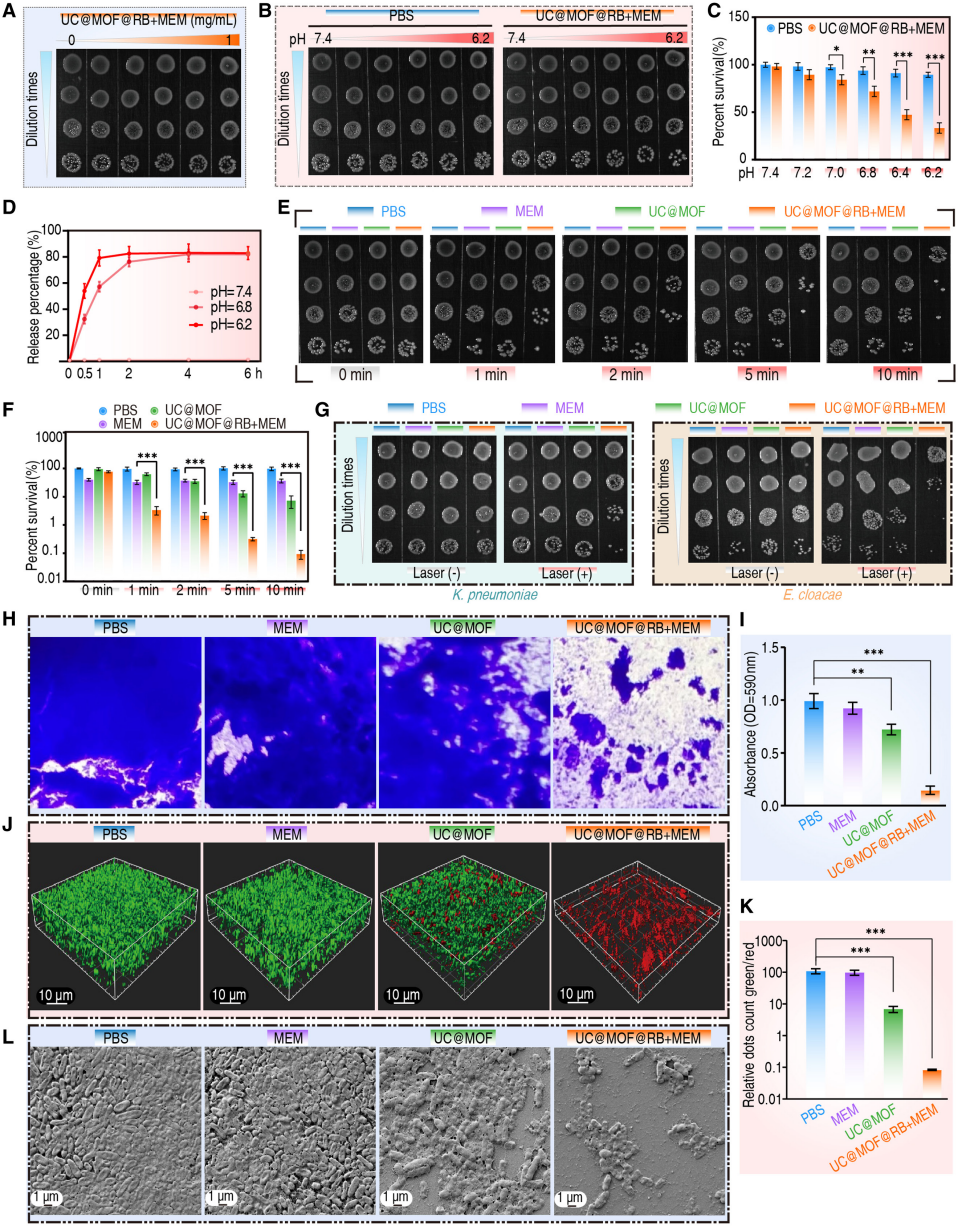
The in vitro antibacterial and anti-biofilm efficacy of nanomaterials. (A) Images of CREC colonies on LB agar plates following incubation with gradient concentrations of UC@MOF@RB+MEM at pH 7.4. (B) Images of bacteria colonies on LB plates after treatment with PBS and UC@MOF@RB+MEM under various pH conditions, and (C) the corresponding survival rate of CREC. (D) Release profile of MEM from UC@MOF@RB+MEM in media with different pH values. (E) Images of colonies on LB agar plates following exposure to PBS, MEM, UC@MOF, or UC@MOF@RB+MEM, with 980-nm laser irradiation (200 mW/cm^2^) for varying durations (0 to 10 min) at pH 6.8, and (F) the corresponding survival rate of CREC treated with different conditions. (G) Images of CRKP (left panel) and CRECL (right panel) colonies on LB agar plates following incubation with various substances at pH 6.8, with or without NIR irradiation (200 mW/cm^2^ for 5 min). (H) Optical microscopy images of CV-stained CREC biofilm following treatment with various substances combined with laser explosion (200 mW/cm^2^ for 30 min), and (I) the corresponding quantitative analysis histograms were generated at a wavelength of 590 nm. (J) Three-dimensional images of CREC biofilm subjected to various treatment conditions. Live bacteria were labeled green by SYTO 9, and dead bacteria were labeled red by PI (scale bar, 10 μm), and (K) quantitative analysis of in vitro anti-biofilm effects. (L) SEM images of CREC biofilms following incubation with PBS, MEM, UC@MOF, and UC@MOF@RB+MEM under 980-nm laser irradiation (200 mW/cm^2^, 30 min) (scale bar, 1 μm). All experiments included 3 biological replicates. Intergroup differences were analyzed by the Mann–Whitney *U* test. Data were expressed as mean ± SD. *P < 0.05, **P < 0.01, ***P < 0.001.

To investigate the bactericidal efficacy of this nanomaterial against CRE strains, we selected the clinically relevant and most prevalent carbapenem-resistant *Escherichia coli* (CREC) (Table S1). UC@MOF@RB+MEM showed no bactericidal activity against CREC under neutral conditions (pH 7.4; Fig. 3A). However, as the pH decreased, a progressive reduction in bacterial count was observed, with significant effects noted at pH 7.0 and below (Fig. 3B and C). Further analysis revealed that UC@MOF@RB+MEM did not release MEM at physiological pH (7.4). In contrast, at pH 6.8, 76.2% of MEM was detected within 2 h, while at pH 6.2, 79.2% of MEM was released within just 1 h, reaching approximately 80% in both cases by 4 h (Fig. 3D). Notably, MEM is typically administered via a 30-min infusion protocol in clinical practice, suggesting that the pH-responsive release kinetics observed here may align with extended infusion strategies. While some studies report no significant improvement in overall clinical outcomes with extended infusion durations, others demonstrate enhanced therapeutic efficacy through optimized pharmacodynamic exposure [35,36]. These results demonstrate that the assembled UC@MOF@RB+MEM efficiently releases MEM under acidic conditions, leading to the killing of CREC. Additionally, at a pH of 6.8, upon further exposure to 980-nm NIR, the bactericidal efficacy of UC@MOF@RB+MEM was enhanced, exhibiting a progressive enhancement with increasing duration of exposure. Notably, 10-min irradiation resulted in 99.9% decrease in viable CREC (Fig. 3E and F). To further validate the bactericidal effects, we tested against 2 additional strains, carbapenem-resistant *Klebsiella pneumoniae* (CRKP) and *Enterobacter cloacae* (CRECL) (Table S1). The treatment achieved bacterial killing rates of 95% and 99.5% for CRKP and CRECL, respectively (Fig. 3G). These findings suggest that short-duration PDT acts synergistically with sustained-release MEM to effectively eradicate the CRE strains. This synergistic strategy exhibits potential clinical utility, prompting further investigation into its potential for biofilm eradication.

Of note, we found that UC@MOF@RB+MEM exhibits significant anti-biofilm activity. Crystal violet (CV) staining showed that biofilms in the phosphate-buffered saline (PBS), MEM, or UC@MOF group remained intact and dense. In contrast, noticeable disruption was evident in the UC@MOF@RB+MEM group (Fig. 3H), along with a significant decrease in optical density (Fig. 3I). Live/dead staining showed no red fluorescence, indicating an absence of dead bacteria in both the control and MEM groups. Faint red fluorescence was seen in the UC@MOF group, while the UC@MOF@RB+MEM group showed a strong red signal (Fig. 3J). The green/red fluorescence ratio dropped by 99.9% in the UC@MOF@RB+MEM group, indicating increased bacterial death (Fig. 3K). Further observations using scanning electron microscopy (SEM) showed no difference in biofilm appearance between the MEM group and the control group. In the UC@MOF group, the biofilm appeared thinner, with bacteria exhibiting signs of collapse. The biofilm in the UC@MOF@RB+MEM group displayed extensive structural damage, with most of the bacteria lysed into cellular fragments (Fig. 3L). These results confirm the efficacy of UC@MOF@RB+MEM against CRE infections.

### In vivo antibacterial efficacy of UC@MOF@RB+MEM

**Fig. 4.**
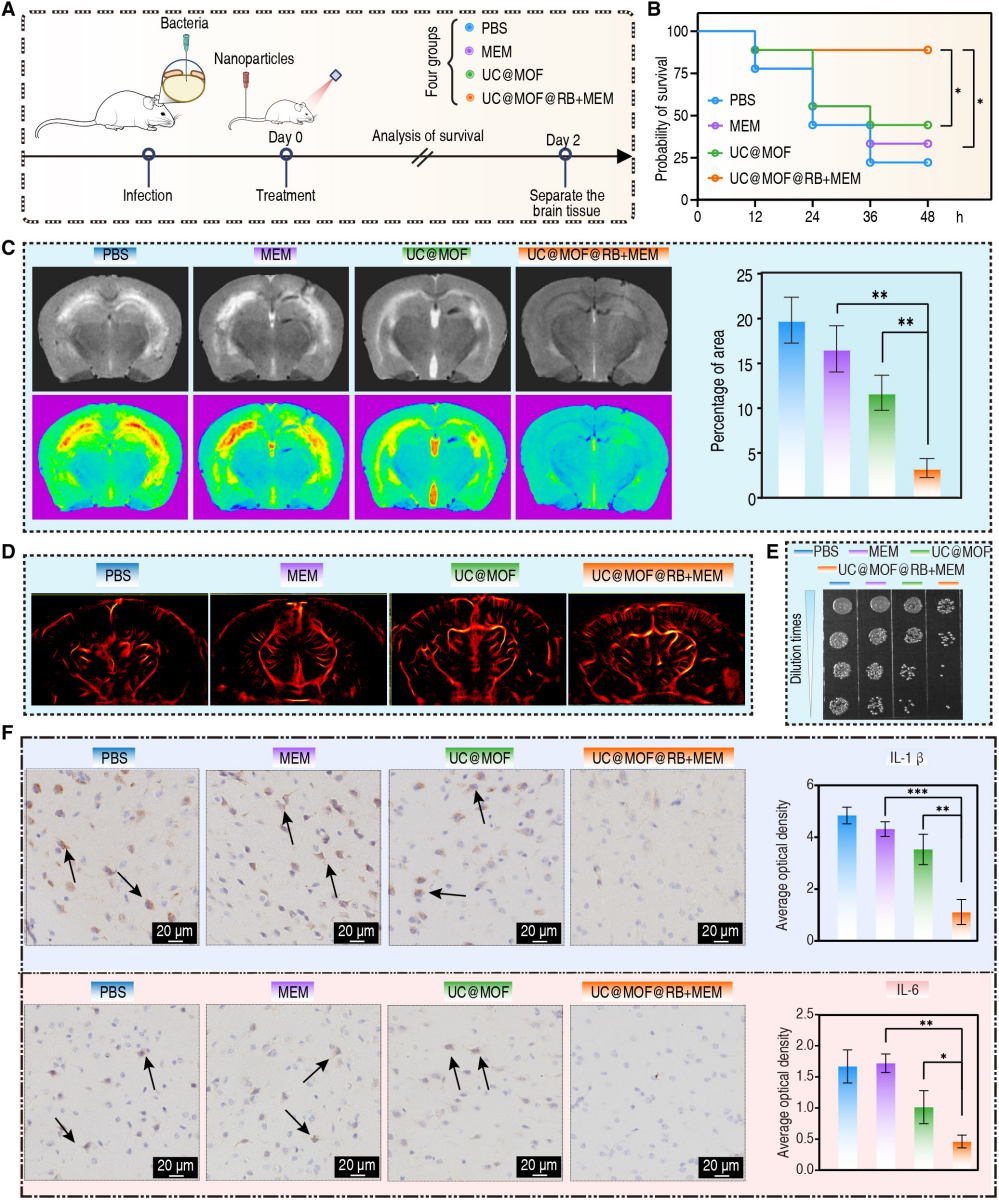
In vivo effect of nanomaterials on treating CNS infections caused by CREC. (A) Schematic illustration of the CNS infection model and experimental timeline for evaluating the therapeutic efficacy of UC@MOF@RB+MEM in treating CNS infections (*n* = 9). Diagram created using BioRender. (B) Survival curves comparing mice treated with PBS, MEM, UC@MOF, or UC@MOF@RB+MEM (log-rank test, *P < 0.05). (C) T2WI of brain tissues from infected mice treated with PBS, MEM, UC@MOF, and UC@MOF@RB+MEM (left panel), and quantitative analysis of the edema area under different treatment conditions (right panel). (D) Ultrasound imaging of the cerebral vessels showing vascular density. (E) Quantitative analysis of bacterial loads in brain tissues from live mice after different treatments. (F) Immunohistochemical staining showing expression levels of IL-1β (upper panel) and IL-6 (lower panel) in brain tissues after different treatments. Arrows point to positive staining for interleukins. Statistical analysis used Mann–Whitney *U* test. Data were presented as mean ± SD. *P < 0.05, **P < 0.01, ***P < 0.001.

Given its strong in vitro antibacterial activity, UC@MOF@RB+MEM was evaluated for treating CNS infections in vivo. We established mouse models of CNS infections by injecting CREC suspension into the intraparenchymal region, followed by tail vein injection of PBS, MEM, UC@MOF, or UC@MOF@RB+MEM, along with 980-nm NIR irradiation (Fig. 4A). Body weight measurements of surviving mice revealed no substantial differences among the 4 groups (Fig. S2). The survival rate for the control group was 22.2%, while the survival rate for mice treated with MEM or UC@MOF was 33.3% and 44.4%, respectively. Importantly, mice receiving UC@MOF@RB+MEM displayed a significant improvement in survival rates, reaching 88.9% (Fig. 4B). Magnetic resonance imaging (MRI) showed considerable brain tissue swelling and shallower local sulci following CREC infection. The edema was particularly pronounced in the cortex, hippocampus, and thalamus, with the hippocampus exhibiting the most substantial changes, characterized by high-signal intensity on T2-weighted imaging (T2WI), indicative of acute inflammation within the brain parenchyma [37,38]. MEM or UC@MOF treatments showed no improvement. However, UC@MOF@RB+MEM treatment significantly reduced brain swelling, with edema almost completely resolved (Fig. 4C, left panel). Quantitative analysis of the edema area further confirmed this observation. The edema area was significantly reduced in the UC@MOF@RB+MEM group, averaging 3.3%, compared to 19.8%, 16.6%, and 11.7% in the PBS, MEM, and UC@MOF groups, respectively, indicating a marked reduction in brain edema (Fig. 4C, right panel). Meanwhile, we performed ultrasound imaging of the cerebral vessels using multi-model ultrafast sonography microscopy (MUSM), which revealed a significant decrease in vascular density in the PBS group, particularly in the cortex and thalamus, whereas the UC@MOF@RB+MEM group showed noticeable improvement, with especially enriched vasculature in the cortex (Fig. 4D). Assessment of cerebral blood flow velocity revealed no significant differences among the 4 groups; it is plausible that severe inflammation and microvascular blockade in the PBS, MEM, and UC@MOF may have induced a compensatory increase in blood flow (Fig. S3A) [39,40]. Further examination of bacterial loads within brain tissue demonstrated a reduction in bacteria following treatment. Mice treated with UC@MOF@RB+MEM showed a significantly greater reduction in bacterial load compared to other groups (Fig. 4E). Histopathological analysis showed that mice receiving MEM still displayed severe microvascular bleeding. A slight alleviation was observed in the UC@MOF-treated group, while such pathological damage was obviously diminished in mice subjected to UC@MOF@RB+MEM treatment (Fig. S3B, upper panel). Cerebellar sections revealed consistent therapeutic effects, with UC@MOF@RB+MEM significantly attenuating microvascular hemorrhage compared to untreated controls (Fig. S3B, lower panel). Furthermore, immunostaining of brain sections showed increased expression levels of interleukin-1β (IL-1β) (Fig. 4F, upper panel) and IL-6 (Fig. 4F, lower panel) in mice treated with MEM, which were comparable to those observed in the control group. Treatment with UC@MOF decreased IL-1β and IL-6 by 27.0% and 39.2%, while UC@MOF@RB+MEM therapy resulted in significant 76.9% and 72.2% reduction of both inflammatory markers. Immunostaining of cerebellar tissues revealed parallel decreases in IL-1β and IL-6 expression, demonstrating consistent anti-inflammatory effects (Fig. S3C). To evaluate the therapeutic effects on neurological functions, behavioral assessments were conducted. In open-field tests, untreated CNS-infected mice, as well as those treated with MEM or UC@MOF, showed increased occupancy in the central zone. In contrast, mice treated with UC@MOF@RB+MEM exhibited edge-preference locomotion patterns, a behavior similar to that of healthy controls (Fig. S4A). Maze experiments further demonstrated that infected mice displayed reduced mobility and decreased exploratory frequency, both of which were improved following treatment with UC@MOF@RB+MEM (Fig. S4B).

Although intravenous MEM is the established first-line treatment for CNS infections, our data demonstrate complete therapeutic failure against emerging carbapenem-resistant strains. UC@MOF@RB+MEM overcomes this limitation by combining MEM with PDT, restoring bacterial susceptibility to MEM while avoiding the prolonged timelines and exorbitant costs of new antibiotic development. This dual-action approach establishes a clinically viable strategy for drug-resistant CNS infections.

### The efficiency of UC@MOF@RB+MEM crossing the BBB

Overcoming the BBB remains a significant challenge in the treatment of CNS infections [41,42]. Here, we presented the specific mechanism through which UC@MOF@RB+MEM crosses the BBB. One of the key factors that enable nanomaterials to cross the BBB is their size. Nanoparticles with dimensions between 10 and 100 nm are particularly advantageous for BBB penetration while minimizing rapid renal clearance [43,44]. The UC@MOF@RB+MEM nanoparticles, with an average size of approximately 50 nm, fall within this optimal range (Fig. 2A, B, and K). Another key parameter influencing the passage of nanomaterials across the BBB is surface charge. The endothelial cells of the BBB exhibit a negative charge due to their higher content of proteoglycans [45,46]. The zeta potential of UC@MOF@RB+MEM was +33.1 mV, indicating a high cationic charge density (Fig. 2J). This positive surface charge promotes electrostatic interactions with the negatively charged luminal membrane of brain endothelial cells and is likely to enhance adsorption and transport to the brain.

**Fig. 5.**
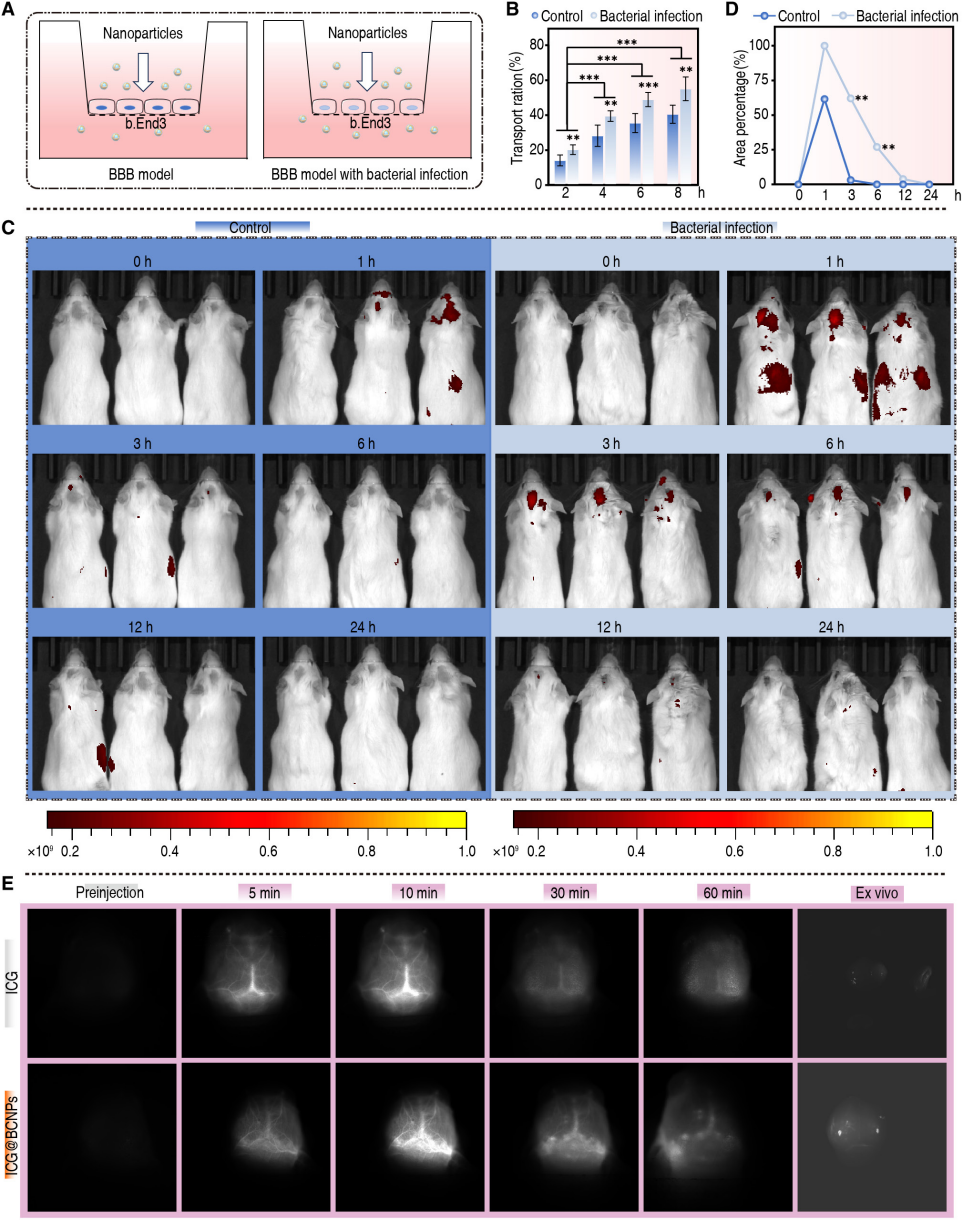
The efficiency of nanomaterials in traversing the BBB. (A) The schematic diagram illustrates the construction of an in vitro BBB model, depicting the differences in UC@MOF@RB+MEM crossing the BBB, and (B) the transport ration of UC@MOF@RB+MEM crossing the BBB within the specified time, in both infected and noninfected models (*n* = 3). (C) Imaging of pharmacokinetics for UC@MOF@RB+MEM in infected and noninfected mice within the first 24 h, and (D) comparison of fluorescence intensity in the brain across different time points. The value of the infected group at 1 h was 100% (*n* = 9). (E) Distribution profile of UC@MOF@RB+MEM in the brain tissue of infected mice. Statistical analysis used Mann–Whitney *U* test. The data are presented as the mean ± SD. **P < 0.01, ***P < 0.001.

Moreover, the increased permeability of the BBB during CNS infections markedly enhanced the efficiency with which UC@MOF@RB+MEM crosses cerebral tissue. During the process of neuroinflammation, inflammatory factors like tumor necrosis factor-α (TNF-α) and IL-1β reduce the expression of tight junction proteins among vascular endothelial cells, thus increasing BBB permeability [47–49]. Here, we presented findings. As illustrated in Fig. 5A, the analysis of nanoparticle permeability using a 2-compartment transwell system showed a significant increase in permeability when cells were cultured in the presence of CREC. Specifically, in the absence of bacteria, only 14.1% of UC@MOF@RB+MEM was detected in the lower chamber after 2 h; this percentage gradually increased to 40.5% by 8 h. When cells were cocultured with CREC, the permeability at 8 h increased by 14.6 percentage points compared to the sterile control (from 40.5% to 55.1%; Fig. 5B). Next, we administered UC@MOF@RB+MEM to mice via tail vein injection and monitored its spatiotemporal distribution using live imaging technology. After 1 h, a substantially higher accumulation of UC@MOF@RB+MEM (indicated by red signals) was observed in the brains of infected mice compared to healthy controls, with most nanoparticles being cleared by 12 h. Simultaneously, a strong red signal was detected in other organs of infected mice at 1 h; however, this signal diminished before 3 h (Fig. 5C and D). Additionally, indocyanine green (ICG) was incorporated into UC@MOF@RB+MEM (ICG@BCNPs) for tracing its accumulation within brain. As shown in Fig. 5E, a stronger signal was observed within inflammatory lesions of the brain 10 min after the injection of ICG@BCNPs, followed by a gradual decrease as ICG@BCNPs were cleared from the body. These results indicate that UC@MOF@RB+MEM is capable of crossing the BBB and targeting inflammatory tissues, demonstrating its potential for the treatment of CNS infections.

### Antibacterial and anti-biofilm mechanism of UC@MOF@RB+MEM

**Fig. 6.**
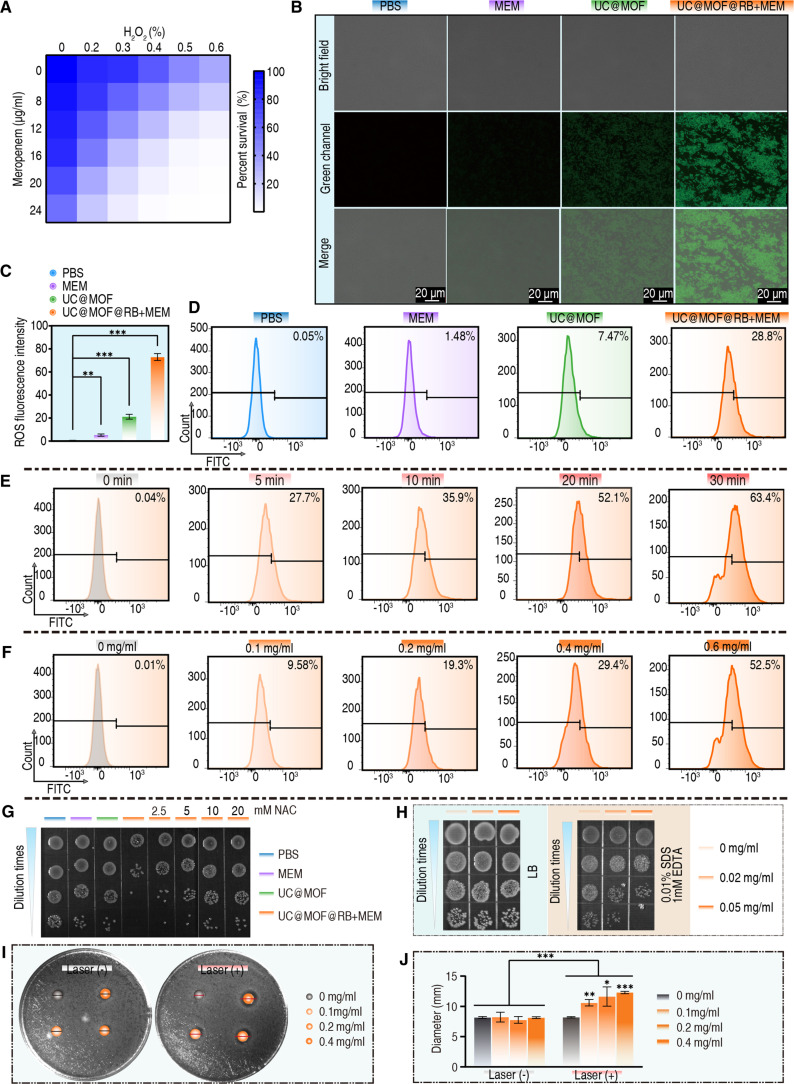
Synergistic effect of ROS and MEM. (A) Survival rate of CREC exposed to various concentrations of MEM in combination with H_2_O_2_. (B) ROS levels in CREC following treatment with PBS, MEM, UC@MOF, or UC@MOF@RB+MEM combined with laser explosion in pH 6.8 media (200 mW/cm^2^, 5 min), and (C) corresponding fluorescence intensity histogram. (D) Quantification of ROS levels under different treatment conditions via flow cytometry. (E) Influence of irradiation time on ROS levels. (F) Impact of UC@MOF@RB+MEM concentration on ROS levels. (G) Images of CREC colonies cultivated on LB plates following incubation with UC@MOF@RB+MEM and NAC. (H) Images of CREC colonies on LB plates with or without SDS and EDTA, following treatment with UC@MOF@RB+MEM under NIR irradiation (200 mW/cm^2^, for 5 min) in pH 7.4 buffer. (I) Influence of UC@MOF@RB+MEM on the MEM inhibition zone in the presence of laser irradiation, and (J) the diameter of the corresponding inhibition zone. All experiments were performed in triplicate biological replicates, and statistical significance was determined using the Mann–Whitney *U* test. Bar graphs depict mean ± SD. *P < 0.05, **P < 0.01, ***P < 0.001.

It is well established that ROS enhances the susceptibility of multidrug-resistant bacteria to certain antibiotics [50]. To this end, we demonstrate that the combination of ROS and MEM is capable of eliminating CRE biofilms rapidly and effectively. Hydrogen peroxide (H_2_O_2_) is one of the most extensively studied ROS in biomedical research. Due to its easily controlled concentration and relatively high stability under experimental conditions, H_2_O_2_ was selected as the ROS donor in this study. As shown in Fig. 6A, compared to treatment with MEM or H_2_O_2_ alone, their combination exhibits a more pronounced elimination on CREC, confirming the synergistic interaction between ROS and MEM.

ROS oxidize 2′,7′-dichlorofluorescein diacetate (DCFH-DA) to produce green fluorescent dichlorofluorescein (DCF), measurable under a fluorescence microscope as an indicator of ROS production [51]. As expected, minimal green fluorescence was observed in cells treated with PBS or MEM alone. In contrast, bacteria exposed to UC@MOF or UC@MOF@RB+MEM emitted green fluorescence, with the UC@MOF@RB+MEM group exhibiting notably stronger fluorescence (Fig. 6B). The fluorescence intensities for the 4 groups were 0.56, 5.2, 21.2, and 72.9, respectively (Fig. 6C). Flow cytometry analysis indicated that only 1.48% of bacteria exposed to free MEM exhibited green fluorescence, in stark contrast to the 28.8% observed in the UC@MOF@RB+MEM group (Fig. 6D). In the UC@MOF@RB+MEM group, longer exposure to NIR increased fluorescence from 27.7% for 5 min to 63.4% for 30 min (Fig. 6E). Similarly, increasing the concentration of UC@MOF@RB+MEM led to a higher number of bacteria displaying green fluorescent signals, with a notable 52.5% at a concentration of 0.6 mg/ml (Fig. 6F). Moreover, when N-acetylcysteine (NAC), a ROS inhibitor, was added to the UC@MOF@RB+MEM group, the bacterial survival percentage increased, and this increase was more pronounced with higher concentrations of NAC (Fig. 6G). Live/dead staining further confirmed that UC@MOF@RB+MEM induced significant bacterial death, which was attenuated by NAC (Fig. S5). These findings demonstrate that PDT and MEM have synergistic effects and effectively eradicate CREC.

Numerous studies have established that ROS are capable of disrupting bacterial cell membranes and biofilms, enhancing the penetration of drugs into the biofilm matrix, and thereby enabling more effective action against the resident bacteria [52–55]. As depicted in Fig. 3I, UC@MOF@RB+MEM demonstrated a significant ability to disrupt CREC biofilms. Further experiments revealed that bacteria treated with UC@MOF@RB+MEM exhibited a reduced survival rate upon exposure to sodium dodecyl sulfate (SDS), indicating an instability in the bacterial membrane and a diminished tolerance to SDS [56] (Fig. 6H). The primary mechanism contributing to carbapenem resistance in Enterobacteriaceae is the production of carbapenemases [57]. Here, we found that ROS diminished the activity of carbapenemase. Specifically, the control group without nanomaterials showed a diameter of inhibition zone for MEM at approximately 8 mm, indicating that the CREC produced carbapenemase [58,59]. In the absence of laser irradiation, the inhibition zone for MEM remained unchanged despite increasing concentrations of UC@MOF@RB+MEM. Importantly, upon exposure to laser, the inhibition zone for MEM expanded, suggesting a reduction in carbapenemase activity (Fig. 6I and J). Collectively, these findings suggest that the therapeutic efficacy against CREC biofilm-associated infections arises from the combined effects of PDT and antibiotics, highlighting the clinical potential of this integrated strategy.

### The neuroprotective efficacy of UC@MOF@RB+MEM

Bacterial lipopolysaccharide (LPS) activates the immune system, triggering neuroinflammatory responses [60,61]. To mitigate this adverse effect, Zn^2+^ was encapsulated into the nanomaterials, which was aimed at providing UC@MOF@RB+MEM with anti-inflammatory functionality (Fig. 4F).

**Fig. 7.**
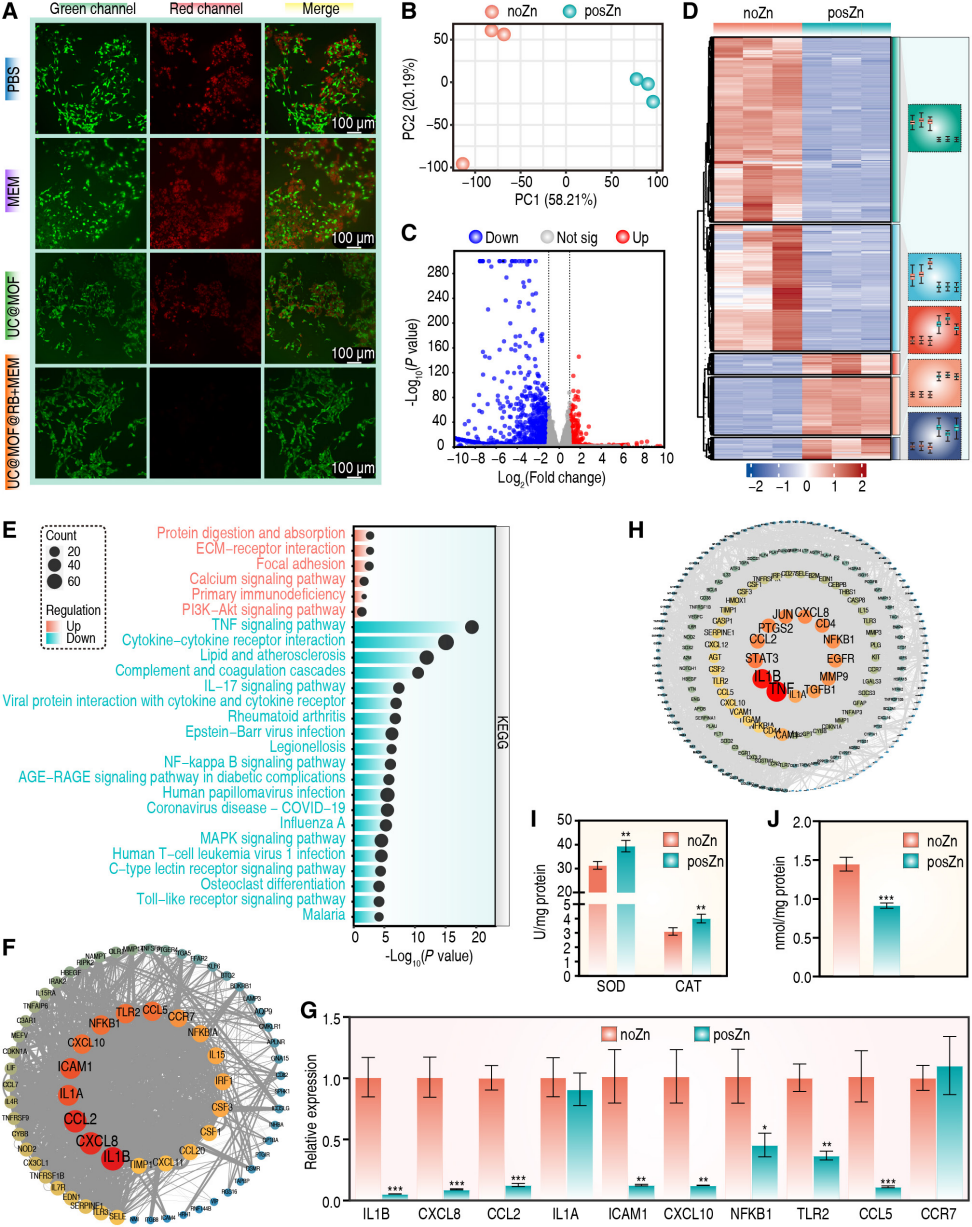
Neuroprotective efficacy of nanomaterials in treating CNS infections. (A) Representative fluorescence images of SH-SY5Y cells treated with PBS, MEM, UC@MOF, or UC@MOF@RB+MEM. Green fluorescence indicates live cells, and red fluorescence indicates dead cells. Scale bar, 100 μm. (B) PCA demonstrates the clustering and dispersion of samples. Each point represents an individual sample, with different colors indicating distinct groups. (C) Volcano plots illustrate the fold change and significance of genes. (D) Heatmap showing the expression level of DEGs, with a color gradient from blue (low expression) to red (high expression). (E) KEGG pathway analyses for both up-regulated and down-regulated DEGs. (F) PPI network demonstrating the hub genes involved in anti-inflammation. The node size represents the number of connections (degree), and the node color represents betweenness centrality. Blue indicates low betweenness centrality, and red indicates high betweenness centrality. (G) qRT-PCR for expression of hub genes. (H) PPI network illustrating the hub genes associated with antioxidation, with node size indicating degree and color representing betweenness centrality (blue indicates low betweenness centrality; red indicates high betweenness centrality). (I) Determination of SOD and CAT activities. (J) Measurement of MDA content. Three biological replicates were conducted, and intergroup difference was assessed by the Mann–Whitney *U* test. Data are presented as mean ± SD. *P < 0.05, **P < 0.01, ***P < 0.001.

Bacterial infection caused significant death of SH-SY5Y neuroblastoma cells. As depicted in Fig. 7A, treatment with MEM had no effect on cell viability, whereas the cell condition improved following treatment with UC@MOF. Notably, upon treatment with UC@MOF@RB+MEM, we observed a significant reduction of the red fluorescence signal, which is associated with cell death. Quantitative analysis revealed that the cell survival rates increased to 90.6% compared to the control group (Fig. S6B). A similar trend was observed in bEnd.3 cells (Fig. S6A and B). These results indicate the protective effect of UC@MOF@RB+MEM on neural cells.

To investigate the role of Zn^2+^ in reducing proinflammatory responses, we stimulated SH-SY5Y cells using LPS [62,63]. Two groups were tested: one with Zn^2+^ (posZn) and one control without Zn^2+^ (noZn). Both groups underwent simultaneous laser irradiation to generate ROS and induce oxidative stress. After a 12-h treatment, the cells were harvested for transcriptomic sequencing analysis. Hierarchical clustering revealed significant differences in transcriptional profiles between the 2 groups, each forming distinct clusters (Fig. S7A). Principal components analysis (PCA) confirmed the distinctive impact of Zn^2+^ on intracellular transcriptional network (Fig. 7B). Using an absolute log_2_(fold change) ≥1 and an adjusted *P* value <0.05 as criteria, we identified 1,481 differentially expressed genes (DEGs), of which 1,117 were down-regulated and 364 were up-regulated (Fig. 7C). A heatmap illustrated specific expression patterns of these DEGs across samples (Fig. 7D). We subsequently conducted Gene Ontology (GO) functional enrichment and Kyoto Encyclopedia of Genes and Genomes (KEGG) pathway analyses on both up-regulated and down-regulated DEGs. For up-regulated genes, GO analysis indicated involvement in biological processes (BPs) including nerve conduction, synaptic transmission, regulation of blood pressure, phosphatidylinositol 3-kinase (PI3K) signaling, and detection of mechanical stimulus involved in sensory perception of sound (Fig. S7B, left panel). KEGG pathways were significantly enriched in calcium signaling pathway and PI3K–Akt signaling pathway (Fig. 7E). Conversely, down-regulated genes were primarily associated with immune regulation and inflammatory responses (Fig. S7B, right panel), with KEGG analysis highlighting significant enrichment in TNF signaling pathway, cytokine–cytokine receptor interaction, complement and coagulation cascades, IL-17 signaling pathway, nuclear factor κB (NF-κB) signaling pathway, mitogen-activated protein kinase (MAPK) signaling pathway, C-type lectin receptor signaling pathway, and Toll-like receptor signaling pathway (Fig. 7E). NF-κB serves as a core regulator in the inflammatory response, with increasing evidence indicating that Zn negatively modulates NF-κB signaling. Zn prevents NF-κB’s nuclear translocation and inhibits inflammation by modulating the IκB kinase complex that phosphorylates NF-κB inhibitory protein [64]. The MAPK pathway is also vital in inflammation. Zn inhibits cyclic nucleotide phosphodiesterase (PDE), resulting in increased guanosine 3′,5′-monophosphate (cGMP) levels that activate protein kinase A (PKA). PKA phosphorylates Raf-1, subsequently inactivating MAPK and NF-κB signaling [65]. These findings demonstrate the positive influence of Zn^2+^ on inflammatory and anti-inflammatory signaling pathways.

To clarify the molecular mechanisms underlying the anti-inflammatory effects of Zn^2+^, we intersected 200 inflammation-related genes from the database with 1,481 DEGs, identifying a total of 74 genes (Fig. S7C). Most displayed down-regulation, notably, Toll-like receptor TLR2, IL-1 receptor-associated kinase IRAK2, and pro-inflammatory cytokines IL1B, TNFRSF9, CXCL8, ICAM4, CCL2, CCL20, and ICAM1 (Fig. S7D and E). We subsequently constructed a protein–protein interaction (PPI) network for these genes; Table S2 listed the degree of all 74 genes. Notably, IL1B, CXCL8, and CCL2, marked in red, presented the highest betweenness centrality values (Fig. 7F). The top 10 hub genes exhibiting the highest betweenness centrality values were further examined through quantitative reverse transcription polymerase chain reaction (qRT-PCR); results were consistent with the transcriptomic data. In the presence of Zn^2+^, transcription levels of IL1B, CXCL8, CCL2, ICAM1, CXCL10, NFKB1, TLR2, and CCL5 significantly decreased except for IL1A and CCR7 (Fig. 7G). These findings reveal that Zn^2+^ suppressed the expression of inflammation-related genes, inhibiting activation of key pathways like MAPK and NF-κB, and thereby mitigating LPS-induced inflammatory responses.

It has been demonstrated that NF-κB influences oxidative stress independently or through various pathways [66]. Inhibiting the NF-κB pathway may reduce oxidative stress [67,68]. We identified 233 DEGs related to oxidative stress through intersection analysis (Fig. S7F and G). Figure S7H illustrates that these genes were predominantly enriched in the TNF signaling pathway, advanced glycation end products–receptor for advanced glycation end products (AGE-RAGE) signaling pathway, IL-17 signaling pathway, MAPK signaling pathway, cytokine–cytokine receptor interaction, NF-κB signaling pathway, and Toll-like receptor signaling pathway. A PPI network was constructed for these genes; Table S3 presented the degree of 233 genes, with TNF, IL1B, STAT3, CCL2, PTGS2, JUN, CXCL8, CD4, NFKB1, and EGFR exhibiting high betweenness centrality (Fig. 7H). Further experiments demonstrated that Zn^2+^ treatment increased levels of antioxidant enzymes superoxide dismutase (SOD) and catalase (CAT) (Fig. 7I) while reducing the oxidative stress biomarker malondialdehyde (MDA) (Fig. 7J). These results suggest that Zn^2+^ exerts an antioxidant effect, reducing the negative effects associated with infection.

In summary, these findings explain the protective mechanism of Zn^2+^ on nerve cells at the molecular level and demonstrate the potential efficacy of UC@MOF@RB+MEM in treating CNS infections.

### The safety and biocompatibility of UC@MOF@RB+MEM in vitro and in vivo

Safety of any therapeutic product is an essential prerequisite for the clinical translation and implementation of nanomedicines [69]. Here, we investigated the safety of UC@MOF@RB+MEM using preclinical in vitro and in vivo models.

**Fig. 8.**
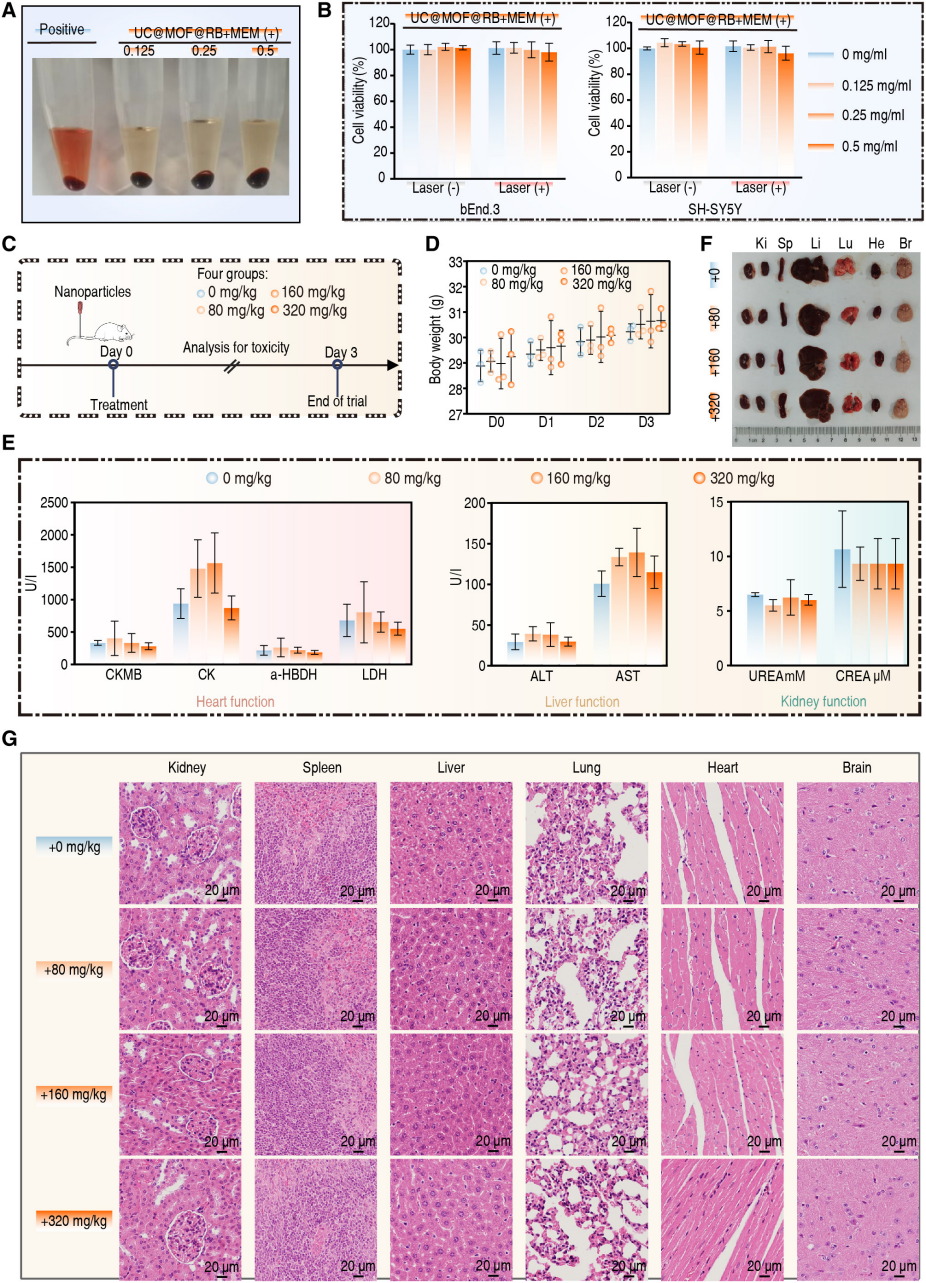
Biosafety of nanomaterials. (A) Hemocompatibility assessment of varying dosages of UC@MOF@RB+MEM. (B) Cytotoxicity response of UC@MOF@RB+MEM on bEnd.3 and SH-SY5Y cell lines. (C) Timeline of animal experiments for evaluating the biosafety of UC@MOF@RB+MEM. (D) Measurement of body weight in mice over a 3-d period following treatment with different concentrations of UC@MOF@RB+MEM. (E) Determination of heart, liver, and kidney function indicators in mice administered various doses of UC@MOF@RB+MEM. (F) Photographs depicting major organs from mice treated with UC@MOF@RB+MEM. Ki, Sp, Li, Lu, He, and Br indicate kidneys, spleen, liver, lungs, heart, and brain, respectively. (G) Corresponding H&E staining images. Three biological replicates were performed, and intergroup differences were evaluated using the Mann–Whitney *U* test. Data are presented as mean ± SD.

The hemolysis assay demonstrated that the red blood cells hemolysis rate remained below 5% even at a UC@MOF@RB+MEM concentration of 0.5 mg/ml, indicating good blood compatibility (Fig. 8A). The cell metabolic activity assay (Cell Counting Kit-8) showed that the viability of both bEnd.3 and SH-SY5Y cells exceeded 95% following a 24-h exposure to UC@MOF@RB+MEM at 0.5 mg/ml. This high viability was observed with or without 980-nm laser irradiation, confirming that our nanomaterials are not cytotoxic for neural cells (Fig. 8B).

In in vivo experiments, mice received various doses of UC@MOF@RB+MEM (0, 80, 160, and 320 mg/kg) via tail vein injection. Their health status was continuously monitored over a 3-d period to assess biocompatibility and potential systemic effects of NPs (Fig. 8C). Daily measurements of body weight indicated that none of the treatments led to significant changes in weight (Fig. 8D). Biomarkers associated with heart function including creatine kinase-MB (CK-MB), creatine kinase (CK), α-hydroxybutyrate dehydrogenase (α-HBDH), and lactate dehydrogenase (LDH), as well as liver function biomarkers such as alanine aminotransferase (ALT) and aspartate aminotransferase (AST), along with kidney function indicators including urea (UREA) and creatinine (CREA), were all within physiological levels, suggesting that NPs did not induce toxicity to the liver, heart, or kidney function (Fig. 8E). Histological examination of major organs, including the kidneys, spleen, liver, lungs, heart, and brain, revealed no notable structural changes of injury compared to the control group (Fig. 8F). Microscopically, no visible pathological changes such as cell necrosis, swelling, hemorrhage, or congestion were observed (Fig. 8G). Specifically, to assess the long-term neuro-safety of the NPs, we established a 14-d observation cohort. Immunohistochemical (IHC) analyses revealed that at the 3-d (Fig. S8A) and 14-d (Fig. S8B) time points, the expression of NueN (a marker for neuronal integrity) in brain tissues remained stable. Moreover, the levels of GFAP (astrocyte activation) and Iba1 (microglial response) exhibited comparable levels to controls, confirming that neither neurodegeneration nor neuroinflammation was induced. These results demonstrate the excellent biocompatibility of UC@MOF@RB+MEM.

## Conclusion

This study developed a biomimetic mineralized coating-modified UCNP system, establishing a charge reversal-driven strategy for crossing the BBB. This system exhibited dual pH-responsive and NIR-activatable characteristics, facilitating precise lesion localization and spatiotemporal-controlled drug release. It achieved synergistic eradication of CRE through short-duration PDT and sustained-release MEM, demonstrating significant clinical relevance and translational potential in addressing multidrug-resistant infections. This study systematically characterized the synthesis process and physicochemical properties of the material, and comprehensively evaluated its therapeutic efficacy and biosafety in cell and animal models. More importantly, transcriptomic analysis provided deep insights into the underlying mechanisms of action, offering a novel therapeutic strategy and theoretical foundation for tackling the clinical challenge of CNS infections. This research plays an important role across many interdisciplinary fields, such as biomimetic mineralized materials, brain disease treatment, and infectious diseases treatment, and has the potential to become a highly influential and cited research achievement.

## Methods

### Apparatus

Centrifugation was performed using a Sigma 3-18KS centrifuge (Sigma, Germany). XRD analysis was conducted using a PANalytical Empyrean diffractometer (PANalytical, Netherlands). TEM imaging was carried out using a JEM-2100F transmission electron microscope (JEOL, Japan). Ultraviolet–visible (UV–vis) spectroscopy was performed with a HITACHI U-3900 spectrometer (HITACHI, Japan). The hydrodynamic diameter and ζ potentials of nanoparticles were determined using a Brookhaven 90PLUS (Brookhaven, USA). Fluorescence spectrum analysis was conducted using a FluroMAX-PLUS fluorescence spectrometer (HORIBA, Japan) with an external 980-nm fiber-coupled infrared laser as the excitation light source.

### Synthesis of UCNP

A modified thermal decomposition method was utilized for synthesizing core–shell structured UCNP. To synthesize NaYF_4_:Yb/Er, 0.58 mmol Y(CH_3_CO_2_)_3_, 0.4 mmol Yb (CH_3_CO_2_)_3_, and 0.02 mmol Er (CH_3_CO_2_)_3_ were added into a 3-necked flask containing 6 ml of oleic acid (OA) and 15 ml of 1-octadecene (ODE). A magnetic stirrer was added, and the temperature was raised to 150 °C, stirring for 40 min. The mixture was then cooled to room temperature under stirring. Subsequently, 10 ml of methanol solution containing NH_4_F (0.4 M) and NaOH (1 M) was added into the 3-necked flask, and the solution was stirred for 1 h at room temperature. The temperature was increased to 110 °C, and the reaction mixture was kept under vacuum for 10 min. The stopcock position was then switched to fill the flask with nitrogen, and the temperature was raised to 300 °C. The reaction mixture was maintained at 300 °C for 1.5 h. After cooling to room temperature, ethanol was used to precipitate the products from the solution. After 3 repetitions of precipitation and dispersion, the products were finally dissolved in 5 ml of cyclohexane. Next, to synthesize NaYF_4_:Yb/Er@NaYF_4_, 0.6 mmol Y(CH_3_CO_2_)_3_ was added into a 3-necked flask containing 6 ml of OA and 15 ml of ODE. The mixture was stirred at 150 °C for 40 min and then cooled to room temperature. Then, 10 ml of methanol solution containing NH_4_F (0.24 M) and NaOH (0.6 M), along with 5 ml of the as-prepared NaYF_4_:Yb/Er, was added to the flask. The subsequent steps were the same as described above. Finally, NaYF_4_:Yb/Er@NaYF_4_ was dissolved in 5 ml of cyclohexane.

### Surface modification

The prepared UCNP underwent a surface OA ligand wash using an acid-washing method to transform it into water-soluble nanoparticles. Specifically, the OA-capped UCNP was dispersed in a solution (2 ml, water/ethanol = 1:1) containing HCl (0.05 M) and then subjected to 10 min of ultrasonication followed by vigorous overnight stirring at room temperature. The OA-free UCNP was collected via centrifugation, washed thrice with acid ethanol (pH <4), and finally dispersed in deionized water. Zinc acetate dihydrate (40 mM) and dimethylimidazole (160 mM) were dissolved in 50 ml of ultra-pure water. The OA-free UCNP was added to dimethylimidazole solution, shaken well, then added to zinc acetate solution, thoroughly mixed, and left for 5 min. Finally, the suspension was centrifuged at 100,000 rpm for 10 min to collect the wrapped up-conversion material. Specifically, the electron microscopy samples were dropped onto ultrathin carbon-coated copper grids and air-dried prior to observation.

### Antimicrobial activity test

CRE strains (one CREC, one CRKP, and one CRECL) were obtained from the First Affiliated Hospital of Sun Yat-sen University. Initially, a single colony of CRE was transferred to a liquid Luria–Bertani (LB) medium and incubated at 37 °C for 16 h. The overnight cultures were centrifuged at 5,000 rpm for 5 min and washed twice with PBS. They were then resuspended to a final cell density of 10^8^ colony-forming units (CFU)/ml using M9 minimal medium (containing 17.1 g/l Na_2_HPO_4_•12H_2_O, 3 g/l KH_2_PO_4_, 1 g/l NH_4_Cl, 0.5 g/l NaCl, 10 mM sodium acetate, 2 mM Mg_S_O_4_•7H_2_O, 0.1 mM CaCl_2_). The pH value was adjusted to meet experimental requirements by varying the amounts of Na_2_HPO_4_•12H_2_O and KH_2_PO_4_. Subsequently, different materials were cocultured with the bacterial suspensions and irradiated with 980-nm light (200 mW/cm^2^) for different times according to experimental requirements, and then incubated at 37 °C for 6 h. Finally, 100 μl of the mixtures was collected for gradient dilution and evenly dispersed on solid LB agar, followed by incubation at 37 °C for bacterial colony counting.

### Biofilm removal test

The CRE-saturated cultures (10^7^ CFU) were pipetted into 96-well or 24-well plates and incubated at 37 °C for 48 h. After biofilm formation, they were treated with PBS, MEM, UC@MOF, UC@MOF@RB+MEM, and NAC, followed by irradiation of 980-nm light (200 mW/cm^2^) for 30 min, and then incubated for another 12 h under static conditions. The suspended medium was discarded, and the biofilms were gently rinsed twice with PBS, ensuring that they were not damaged. For CV staining, 100 μl of 10% methanol was added and fixed for 15 min. After removing the methanol, the biofilms were treated with an equal volume of 0.1% CV for 15 min and then washed thoroughly using double-distilled deionized water for optical microscope observation. Meanwhile, 200 μl of 33% acetic acid was added to each well to solubilize the CV, which was detected at 590 nm by a microplate reader. For fluorescence imaging, the biofilms were treated with SYTO 9 and propidium iodide (PI) for 15 min in the dark, and then a 3-dimensional (3D) model of the bacterial biofilms was captured using a confocal laser scanning microscope (CLSM, Leica TCS SP8 STED 3X). For morphological observation, the biofilms were fixed with 2.5% glutaraldehyde for 30 min at room temperature, followed by dehydration with an ethanol solution concentration gradient (30%, 50%, 70%, 80%, and 95%) for 5 min. Next, the bacteria were resuspended in a 95% ethanol solution, and 5-μl suspensions were transferred to the climbing film. After drying, the samples were coated with gilded film for photography through an SEM (Carl Zeiss Crossbeam 550).

### Measurement of total ROS

The overnight cultures were resuspended with M9 minimal medium to OD_600 nm_ = 0.2 and pipetted into 24-well plates. Different drugs were added to the corresponding groups (PBS, MEM, UC@MOF, and UC@MOF@RB+MEM), and all groups were exposed to 980-nm light at a power density of 200 mW/cm^2^ for 5 min. After 2 h of incubation in a constant-temperature incubator at 37 °C, the mixtures were centrifuged at 5,000 rpm for 5 min and washed twice with PBS. Next, DCFH-DA was introduced to the 4 groups and cocultured for 20 min. Subsequently, the mixture was centrifuged and washed with PBS to remove the excess dye. Finally, the suspensions were transferred to a round-bottom tube for determination of ROS using flow cytometry (Backman Cytoflex) (Olympus, excitation wavelength: 488 nm, emission wavelength: 525 nm) and for fluorescence photography using a fluorescence microscope (Nikon Eclipse Ni-E).

### MEM ELISA assay

The experiment was carried out utilizing a MEM test kit (Cloud-Clone Corp C, CEK946Ge). Firstly, UC@MOF@RB+MEM was added to an M9 medium of different pH (6.2, 6.8, and 7.4) and incubated for 0, 0.5, 1, 2, 4, and 6 h, respectively. Then, 50 μl of each system was taken for detection following the instructions of the enzyme-linked immunosorbent assay (ELISA) kit. Finally, the absorbance of each well was recorded at a wavelength of 450 nm. A linear regression curve of the standard was plotted, with the concentration of the standard and the corresponding absorbance being the horizontal axis and *y* axis, respectively. The concentration of each system in different pH environments was calculated according to the standard curve equation.

### In vivo antibacterial study

All animals received care according to the guidelines in the Guide for the Care and Use of Laboratory Animals. Kunming (KM) female mice (6 to 8 weeks old) were selected for the study. Prior to anesthesia, an overnight culture of CREC was collected, washed 3 times with sterile normal saline, and resuspended to an OD_600 nm_ of 0.1. The mice were anesthetized and injected with 10 μl of CREC suspension (10^6^ CFU) into the cerebral parenchyma to induce the CNS infection model. Stereotaxic coordinates relative to bregma were set at 1.5 mm lateral and 1 to 2 mm posterior [70]. After 24 h, the mice were randomly divided into 4 groups, each consisting of 9 mice: (a) PBS, (b) MEM, (c) UC@MOF, and (d) UC@MOF@RB+MEM. Different drugs were then introduced via the caudal vein. One hour later, the mice were exposed to 980-nm light (200 mW/cm^2^) for 30 min. To determine the survival rate, we observed the number of mice that survived and died after 48 h of treatment. Additionally, the body weight of the surviving mice was weighed and recorded from day 1 to day 3. Brain tissues from live mice were homogenized, diluted with normal saline, and then plated on an LB agar plates at 37 °C for 16 h to determine the count of bacteria in the infected tissue. The visible bacterium was observed and captured.

### Dynamic metabolism of materials in vivo

To observe the dynamic metabolism of nanomaterials in vivo, 12 mice were randomly divided into 2 groups: (a) the PBS group (*n* = 3) and (b) the bacterial infection group (*n* = 9, with 6 of them used for organ imaging). Subsequently, PBS and CREC suspension (10^6^ CFU) were injected intracranially into the 2 groups of mice, respectively. After 24 h, UC@MOF@RB+MEM was introduced via the caudal vein, and then live bioluminescence imaging was implemented at 0, 1, 3, 6, 12, and 24 h. Simultaneously, one mouse randomly chosen from the group of 6 was used to dissect major organs (heart, liver, spleen, lung, kidneys, and brain) for fluorescence observation at each time point.

### In vivo neuroprotection experiment

CNS infection models were established to examine the morphological structure and levels of inflammatory factors in brain tissue. Four groups, each consisting of 9 mice, were randomly assigned as follows: (a) PBS, (b) MEM, (c) UC@MOF, and (d) UC@MOF@RB+MEM. Various drugs were administered through the caudal vein. One hour later, the mice were exposed to 980-nm light at 200 mW/cm^2^ for 30 min. After 48 h of treatment, the surviving mice were euthanized, and their brain tissue was dissected for hematoxylin and eosin (H&E) staining and IHC to evaluate IL-1β and IL-6 levels, followed by imaging with a fluorescence microscope (Nikon Eclipse Ni-E). Furthermore, the surviving mice were subjected to MRI scans (PharmaScan70/16 US) to assess brain injury.

### Tissue reactivity of UC@MOF@RB+MEM

KM mice were randomly divided into 4 groups, each consisting of 3 mice: (a) 0 mg/kg, (b) 80 mg/kg, (c) 160 mg/kg, and (d) 320 mg/kg. The groups received different doses of UC@MOF@RB+MEM via intravenous injection and were monitored for changes in body weight over the course of 3 d. After 72 h, all the mice were anesthetized and euthanized. Blood samples were collected to detect liver, heart, and kidney function indicators. Additionally, major organs, including the brain, heart, lung, liver, spleen, and kidneys, were dissected for observation and H&E staining.

### BBB-crossing model in vitro

The BBB model was established in a 12-well plate with a 12-mm transwell chamber featuring a 0.4-μm polycarbonate membrane (LABSELECT, China). The bEnd.3 cells were seeded at a concentration of 2 × 10^4^ cells/cm^2^ and cultured for 2 d. Cells were not selected for experiments until the transendothelial electrical resistance (TER) of the cell barrier was ≥200 Ω/cm^2^, measured using the World Precision Instruments EVOM2 (Sarasota, FL, USA). Next, UC@MOF@RB+MEM diluted in Dulbecco’s modified Eagle’s medium (DMEM) at a concentration of 1 μg/ml was added into the upper chamber of the transwell plate (blood-side in vivo) and subjected to 50 rpm shaking at 37 °C for 10 h. Subsequently, the upper and lower chamber media were collected at 2, 4, 6, and 8 h, and the contents of UC@MOF@RB+MEM were analyzed for fluorescence intensity. In the case of the bacterial infection model, the CRE cultures were introduced to the upper chamber at a concentration of 10^6^ CFU/ml. After 3 h of incubation, the bacteria suspension was removed, and cells were washed twice with PBS. Then, UC@MOF@RB+MEM was added, and the fluorescence intensity was determined according to the above procedure.

### BBB-crossing test in vivo

ICG was mixed with UC@MOF@RB+MEM and incubated for 6 h, followed by centrifugation to remove unbound ICG. The resulting conjugate was then injected into mice with CNS infections via the tail vein at a dosage of 5 mg/kg. In vivo fluorescence distribution was monitored using a real-time NIR-II imaging system (Yuan Ao Instrument, China). An 808-nm laser beam (Changchun Lighting Co. Ltd., China) was expanded through a lens and coupled to a collimator to achieve uniform illumination. Prior to imaging sessions, the laser power density was measured and calibrated to 10 to 20 mW/cm^2^. NIR-II fluorescence signals were collected utilizing long-pass filters.

### Live/dead cell staining

CRE strains with a concentration of 10^8^ CFU/ml were treated with different solutions (PBS, MEM, UC@MOF, UC@MOF@RB+MEM), exposed to 980-nm light at 200 mW/cm^2^ for 5 min, and incubated for 6 h. SH-SY5Y and bEnd.3 cells (5 × 10^4^ cells per well) were plated and maintained in 24-well culture plates for 24 h. Next, the media were replaced with 1 ml of fresh DMEM media, and 100 μl of the CRE strain suspensions, which had undergone different treatments (PBS, MEM, UC@MOF, and UC@MOF@RB+MEM), was added. The cocultures were maintained at 37 °C for 3 h, after which the cells were washed twice with PBS. Subsequently, the cells were incubated with a solution of calcein-AM and PI (Beyotime, China) at 37 °C for 30 min before being observed and photographed using a fluorescence microscope (Nikon Eclipse Ni-E). Meanwhile, the ratios of live cells (calcein-AM^+^, PI^−^) and dead cells (calcein-AM^−^, PI^+^) were calculated.

### Transcriptional reporter assay

A complete description of the experimental method and transcriptome analysis flow is available in Supplementary Methods.

### Activity assay of SOD and CAT

PBS (0.5 ml) was added to each portion of cells (1 × 10^6^ cells). The cells were then subjected to ultrasonic disruption in an ice-water bath for 5 min (power: 200 W, on/off cycles: 2 s/3 s), followed by centrifugation at 3,000 rpm for 10 min. The supernatant was collected to quantify protein concentration using BCA protein assay kit (Beyotime), and the activities of SOD and CAT were measured using SOD assay kit and CAT assay kit (Nianjing Jiancheng Bioengineering Institute), respectively.

### qRT-PCR

Total RNA was extracted using the MolPure Cell/Tissue Total RNA Kit, and the quantity and quality of the RNA were assessed with a NanoDrop (Thermo Scientific). Reverse transcription of RNA was performed utilizing the Evo M-MLV RT Kit, while real-time fluorescence quantitative PCR was conducted employing the SYBR Green Pro Taq HS qPCR Kit. All experimental procedures were strictly carried out in accordance with the instructions. The detection instrument utilized was the LightCycler 480 II (Roche). The expression level of the gene in the control group was normalized to 1, and the expression levels of the gene in the treatment group were compared with those of the control group. The relevant primers were listed in Supplementary Methods (Table S4).

### Hemocompatibility assay

The hemocompatibility of UC@MOF@RB+MEM was assessed through a hemolysis test. Mouse blood (1 ml) was diluted with 0.9% normal saline solution and centrifuged at 4,000 rpm at 4 °C for 10 min to isolate the erythrocytes. A 10% suspension of the isolated erythrocytes was prepared and mixed with deionized water (positive control) or different concentrations (0.125, 0.25, and 0.5 mg/ml) of UC@MOF@RB+MEM. Following a 4-h incubation at 37 °C, the supernatant was separated by centrifugation at 3,500 rpm for 10 min, and hemolysis was assessed based on color changes.

### Statistical analysis

Statistical significance was assessed using the Mann–Whitney *U* test. All statistical analyses were performed using R (v4.4.2). Data are presented as mean ± SD. *P < 0.05, **P < 0.01, ***P < 0.001; all *P* values are from 2-tailed tests.

## Ethical Approval

All experiments involving animals were conducted in accordance with the ethical policies and procedures approved by the Institutional Animal Care and Use Committee of Sun Yat-sen University (approval no. SYSU-IACUC-2024-B0989).

## Data Availability

Data generated during the current study are available from the corresponding authors upon reasonable request.
