# Supplementary material for: BBB-Crossing Ionizable Upconversion Nanoparticles for Synergistic Therapy of Carbapenem-Resistant Central Nervous System Infection
Source: Research (Wash D C). 2025 Nov 24;8:0946. doi: 10.34133/research.0946 (PMC12641214; doi:10.34133/research.0946)
Supplement: Supplementary 1 — Figs. S1 to S8 Tables S1 to S4 Supplementary Methods [file research.0946.f1.docx]

**BBB-crossing Ionizable Upconversion Nanoparticles for Synergistic Therapy of Carbapenem-Resistant Central Nervous System Infection**


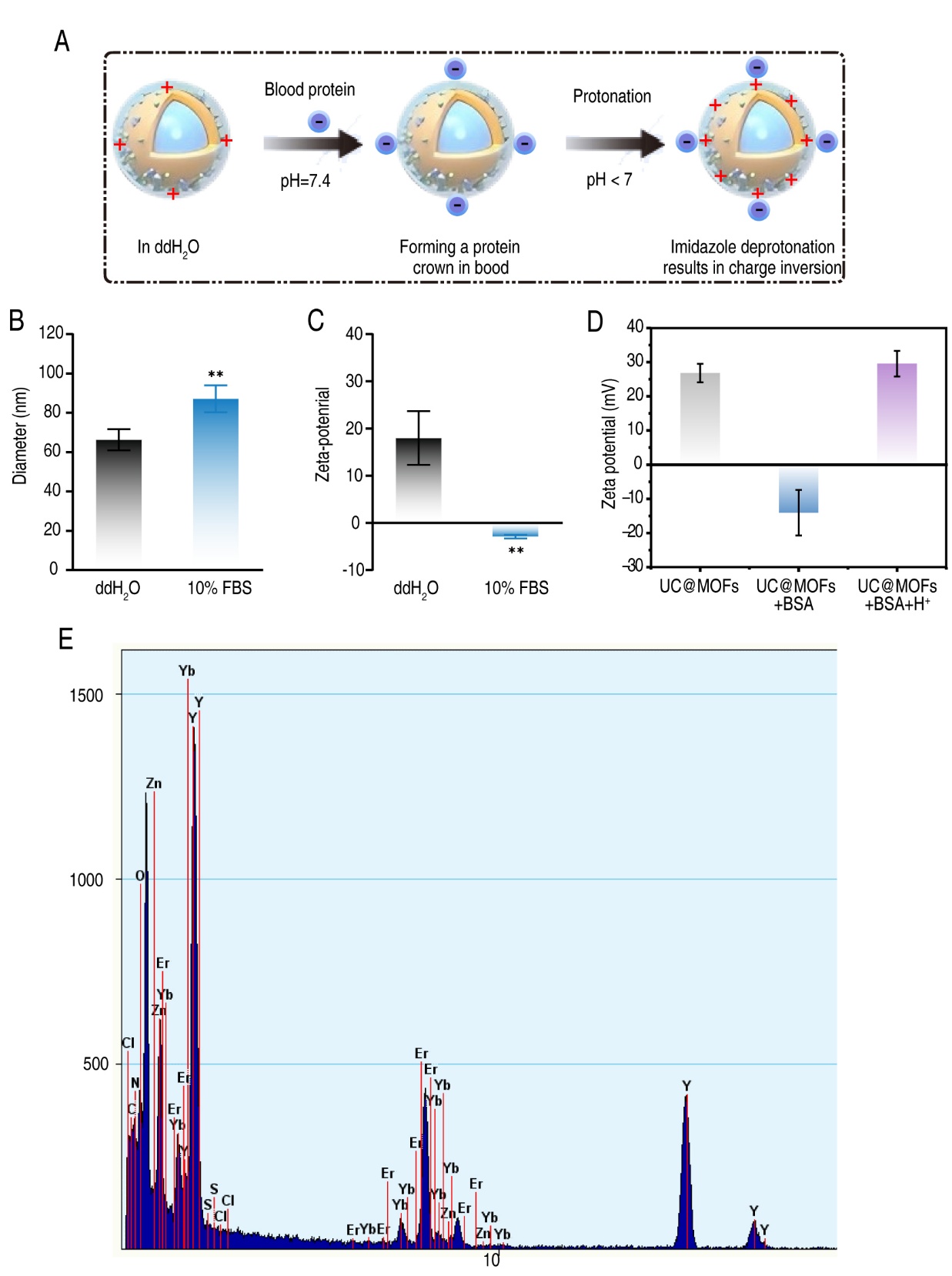


**Fig. S1 Protein-mediated changes in physical properties.** A. Schematic diagram. B. Change of diameter in physical properties. C. Change of surface charge in physical properties. D. zeta potential changes in acidic microenvironments. E. Energy dispersive X-ray (EDX) spectrum of UC@MOF@RB+MEM.


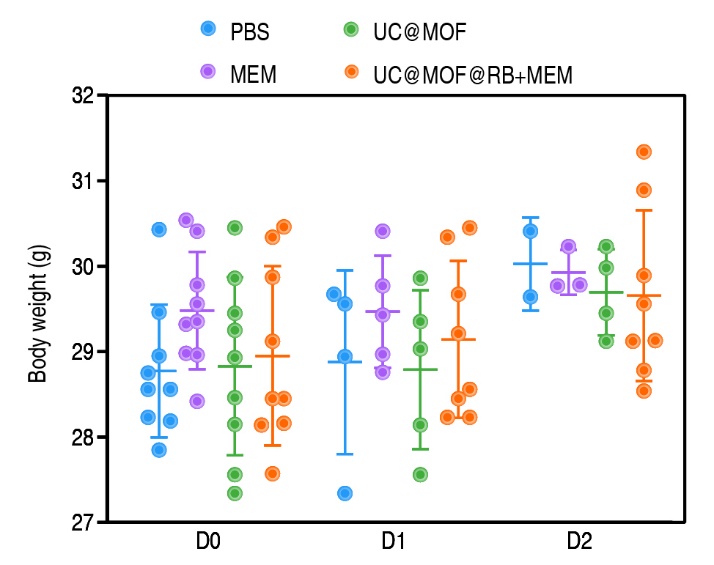


**Fig. S2 Changes in body weight of surviving mice following different treatments.**

**
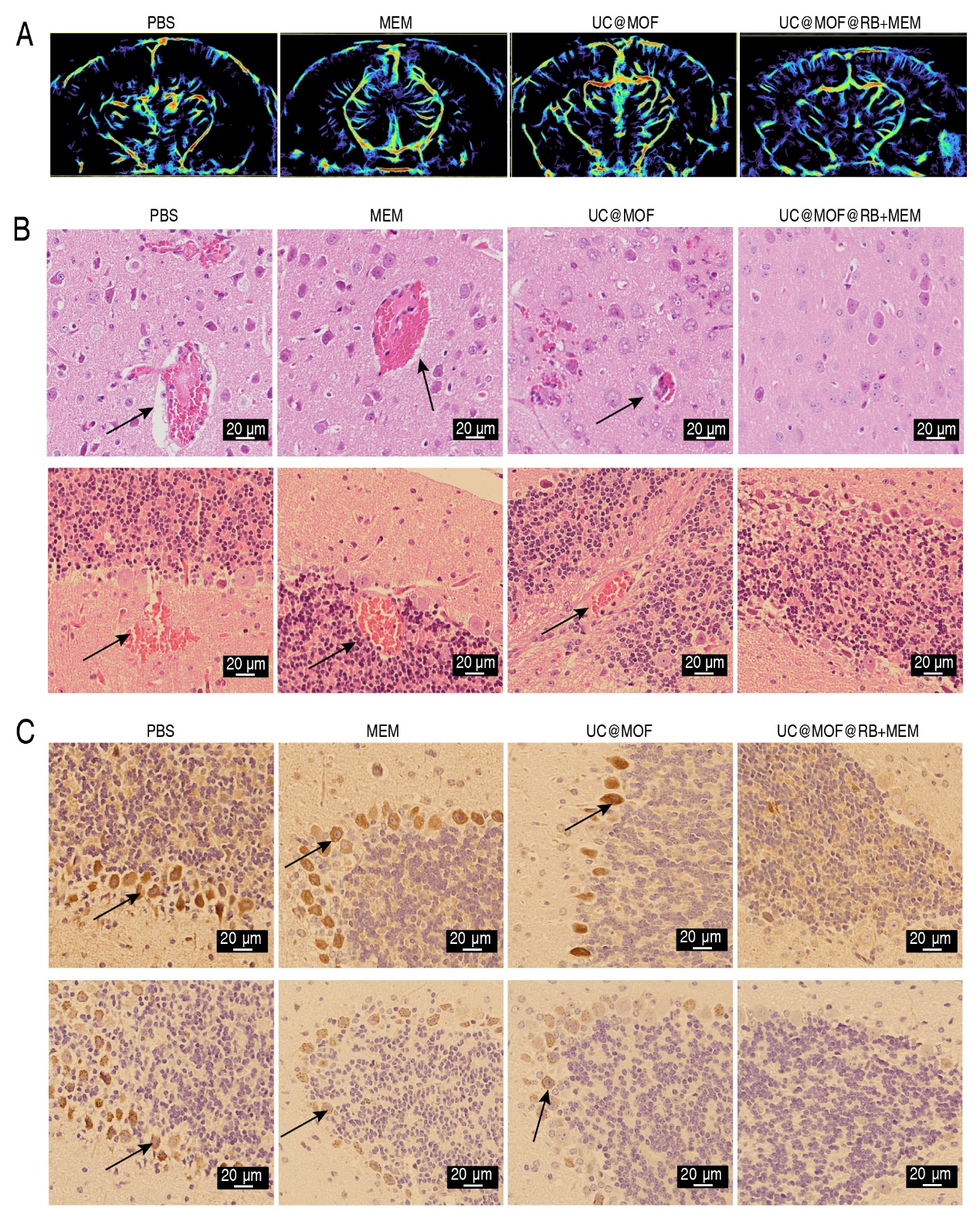
**

**Fig. S3** **Evaluation of cerebrovascular hemodynamics, histopathology, and neuroinflammatory responses.** (A) Ultrasound imaging of the vasculature showing cerebral blood flow velocity. (B) H&E staining illustrating microvascular hemorrhage in cerebral (upper panel) and cerebellar regions (lower panel). Arrows indicate areas of microvascular bleeding. (C) Immunohistochemical staining showing expression levels of IL-1β (upper panel) and IL-6 (lower panel) in cerebellar tissues after different treatments. Arrows highlight positive immunoreactive signals.

**
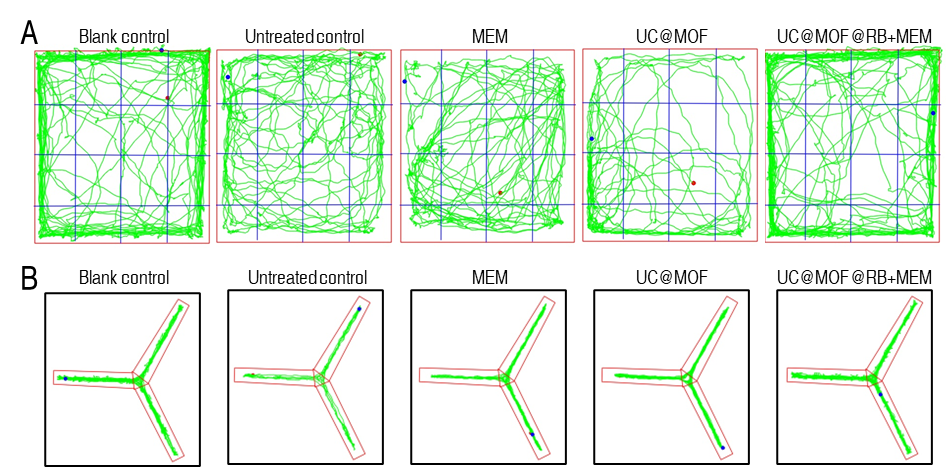
**

**Fig. S4 Neurofunctional assessment.** (A) Open field test comparing spatial preferences. (B) Maze experiments comparing locomotor activity and exploratory behavior.

**
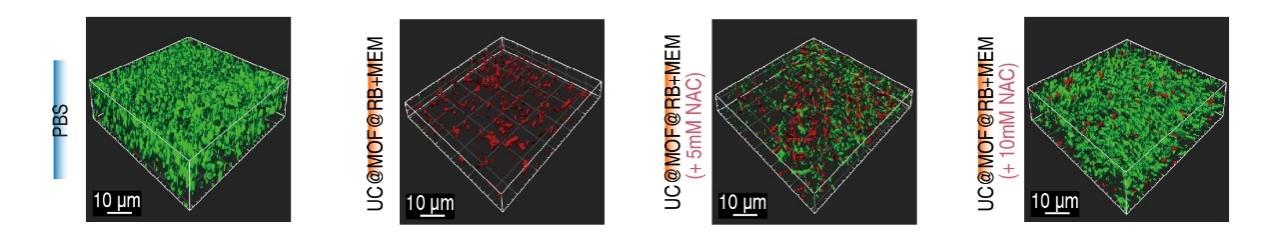
**

**Fig. S5** **3D confocal laser scanning microscope (CLSM) images illustrating the impact of NAC on CREC biofilms**. Viable bacteria were stained green with SYTO 9, while dead bacteria were labeled red by PI. Scale bar: 10 μm.


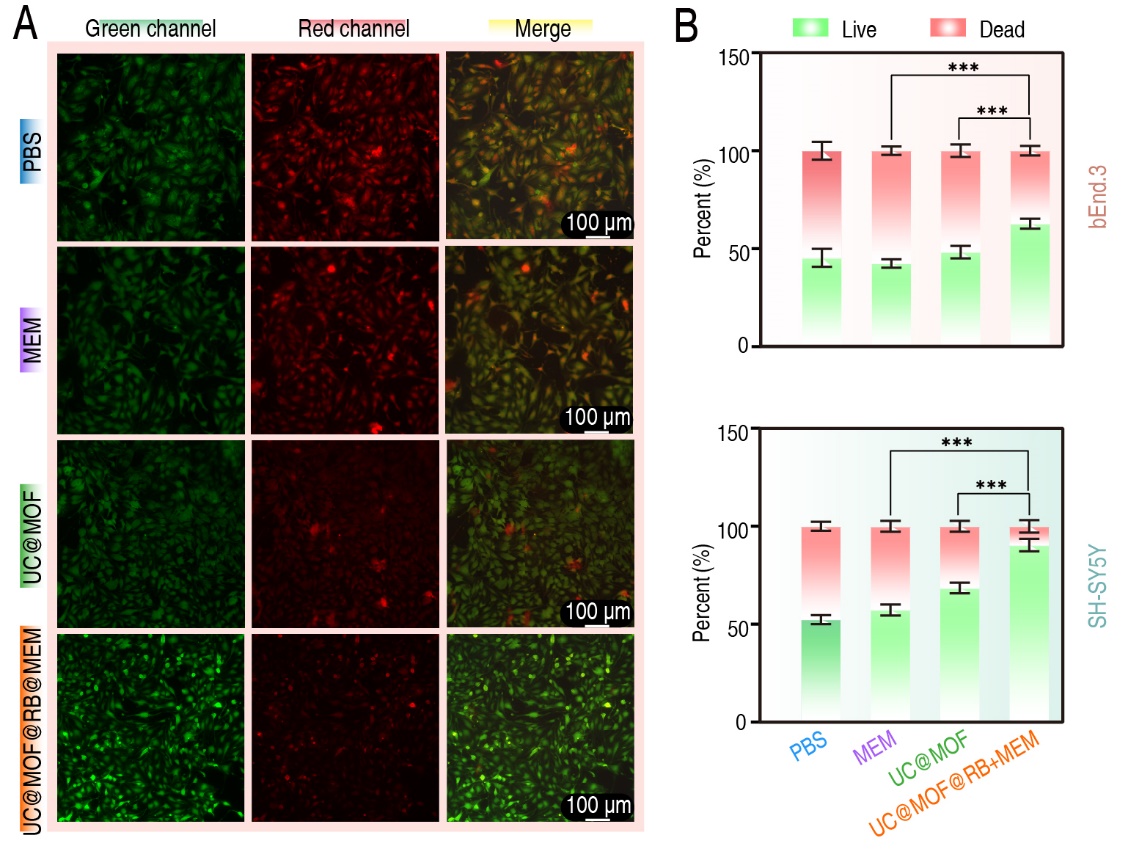


**Fig. S6 Effect of different treatments on cell viability.** (A) Fluorescence images of bEnd.3 cells treated with PBS, MEM, UC@MOF, or UC@MOF@RB+MEM. Green fluorescence indicates live cells, and red fluorescence indicates dead cells. Scale bar: 100 μm, and (B) corresponding live/dead cell ratio for bEnd.3 (upper panel) and SH-SY5Y cells (lower panel).

**
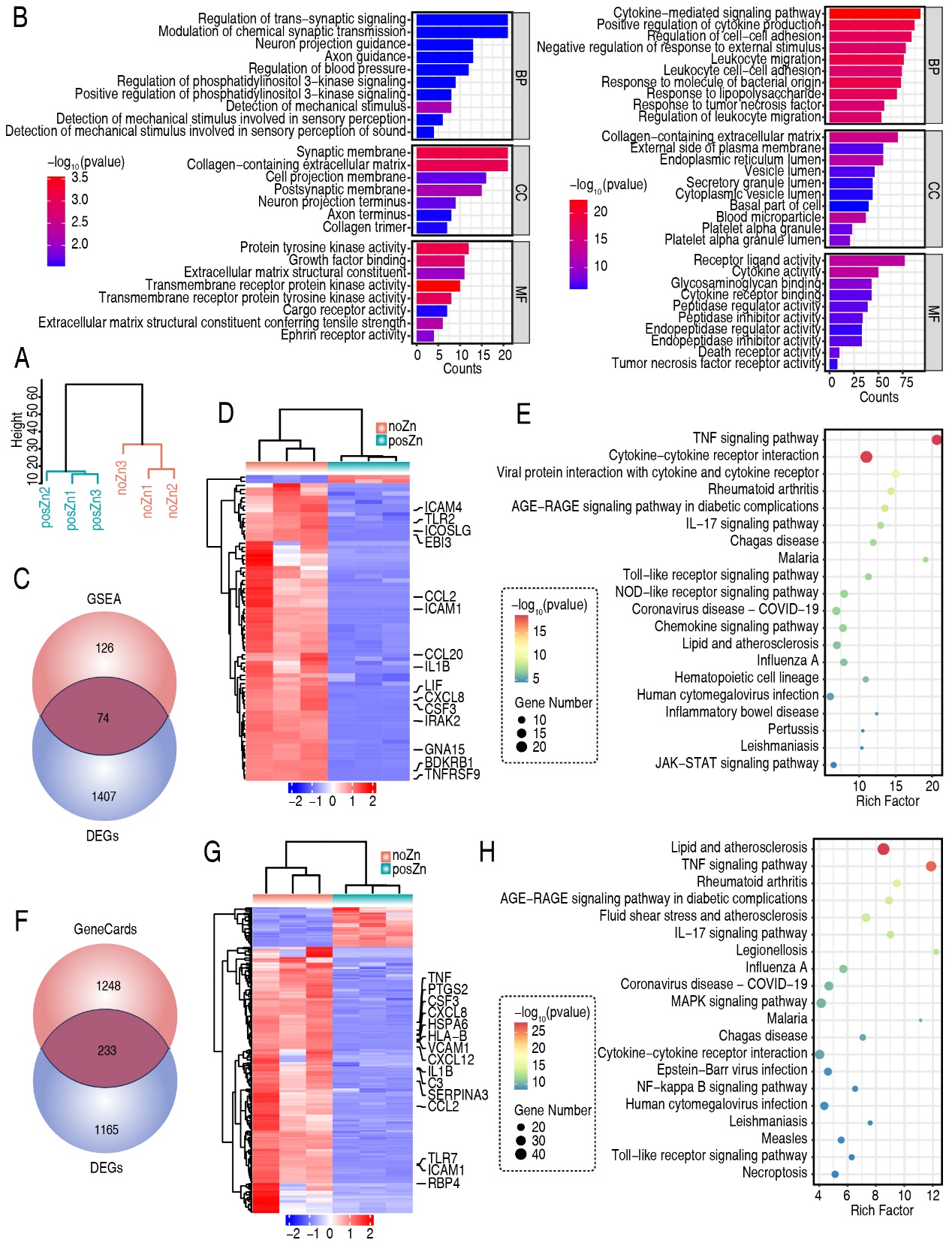
**

**Fig. S7 Transcriptomic responses of SH-SY5Y exposed to Zn^2+^.** (A) Hierarchical clustering dendrogram illustrating the similarity among samples. (B) GO analyses for up-regulated (left panel) and down-regulated (right panel) DEGs. (C) Intersection of DEGs with inflammation-related genes. (D) Heat map displaying the expression level of 74 inflammation-related genes, with a colour gradient ranging from blue (low expression) to red (high expression). (E) KEGG pathway analysis for the 74 inflammation-related genes. (C) Intersection of DEGs with oxidative stress-related genes. (D) Heat map showing the expression level of 233 oxidative stress genes, with a colour gradient from blue (low expression) to red (high expression). (H) KEGG pathway analysis for the 233 oxidative stress-related genes.

**
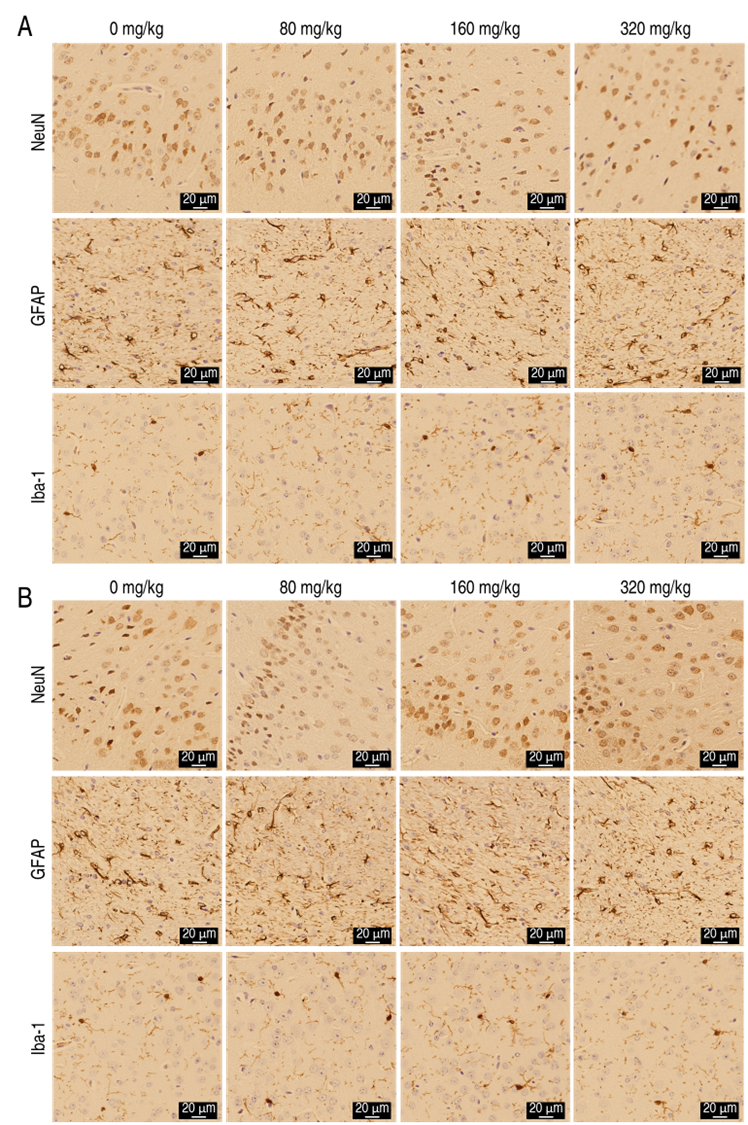
**

**Fig. S8 Assessment of neurological safety following NP treatment.** (A) Short-term (3-day). (B) Long-term (14-day).

**Table S1** **Antibiotics sensitivity of strains**

|  | Antibiotics Strains | CREC | CRKP | CRECL |  |  |
| --- | --- | --- | --- | --- | --- | --- |
|  | Piperacillin/sulbactam | R | R | R |  |  |
|  | Ceftazidime | R | R | R |  |  |
|  | Cefoperazone sulbactam | R | R | R |  |  |
|  | Ceftriaxone | R | R | R |  |  |
|  | Aztreonam | R | R | R |  | Susceptible |
|  | Imipenem | R | R | R |  | **S** |
|  | Meropenem | R | R | R |  |  |
|  | Amikacin | S | S | S |  | Resistant |
|  | Tobramycin | S | R | S |  | **R** |
|  | Ciprofloxacin | R | R | R |  |  |
|  | Levofloxacin | R | R | R |  |  |
|  | Minocycline | R | R | R |  |  |
|  | Tigecycline | S | R | R |  |  |
|  | Colistin | S | S | R |  |  |

CREC: Carbapenem-resistant *Escherichia coli*, CRKP: Carbapenem-resistant *Klebsiella pneumoniae*, CRCL: Carbapenem-resistant *Enterobacter cloacae*.

**Table S2 The degree of inflammation-related genes**

| **Genes** | **Degree** | **Genes** | **Degree** | **Genes** | **Degree** | **Genes** | **Degree** |
| --- | --- | --- | --- | --- | --- | --- | --- |
| IL1B | 49 | SELE | 24 | IRAK2 | 13 | ICOSLG | 4 |
| CCL2 | 46 | TLR3 | 23 | HBEGF | 12 | INHBA | 4 |
| CXCL8 | 46 | EDN1 | 22 | MMP14 | 12 | SPHK1 | 4 |
| ICAM1 | 41 | IL7R | 22 | NAMPT | 12 | GP1BA | 3 |
| IL1A | 41 | SERPINE1 | 22 | OLR1 | 12 | OSMR | 3 |
| CCL5 | 37 | CX3CL1 | 20 | RIPK2 | 12 | PTGIR | 3 |
| CXCL10 | 37 | NOD2 | 20 | PTGER4 | 10 | RGS16 | 2 |
| NFKB1 | 37 | TNFRSF1B | 20 | TNFSF9 | 10 | TAPBP | 2 |
| TLR2 | 37 | CYBB | 18 | FFAR2 | 8 | VIP | 2 |
| CCR7 | 32 | IL4R | 17 | ITGA5 | 8 | HRH1 | 1 |
| NFKBIA | 30 | TNFRSF9 | 17 | BTG2 | 7 | ICAM4 | 1 |
| CSF3 | 29 | CCL7 | 16 | KLF6 | 7 | ITGB8 | 1 |
| IL15 | 29 | CDKN1A | 15 | BDKRB1 | 6 | NMI | 1 |
| IRF1 | 29 | LIF | 15 | LAMP3 | 6 | RNF144B | 1 |
| CCL20 | 27 | C3AR1 | 14 | APLNR | 5 | BEST1 | 0 |
| CSF1 | 27 | IL15RA | 14 | AQP9 | 5 | CHST2 | 0 |
| CXCL11 | 26 | MEFV | 14 | CMKLR1 | 5 | EBI3 | 0 |
| TIMP1 | 26 | TNFAIP6 | 14 | GNA15 | 5 | KCNJ2 | 0 |
|  |  |  |  | CD82 | 4 | PTPRE | 0 |

**Table S3 The degree of oxidative stress-related genes**

| **Genes** | **Degree** | **Genes** | **Degree** | **Genes** | **Degree** | **Genes** | **Degree** | **Genes** | **Degree** |
| --- | --- | --- | --- | --- | --- | --- | --- | --- | --- |
| TNF | 162 | LGALS3 | 52 | IGF2 | 32 | ACHE | 19 | CYP2A6 | 10 |
| IL1B | 154 | SOCS3 | 52 | ISG15 | 32 | CYP1A2 | 19 | MAP2K3 | 10 |
| STAT3 | 116 | MMP1 | 51 | MMP13 | 32 | G6PD | 19 | MT-ND2 | 10 |
| CCL2 | 110 | TNFAIP3 | 51 | PDGFB | 32 | GAD1 | 19 | MT-ND3 | 10 |
| CXCL8 | 110 | CDKN1A | 50 | TAC1 | 31 | HSPA1A | 19 | NRG1 | 10 |
| JUN | 110 | CYBB | 50 | XBP1 | 31 | NCF2 | 19 | TRPA1 | 10 |
| PTGS2 | 110 | GPT | 50 | BMP4 | 30 | NR4A2 | 19 | UCP1 | 10 |
| CD4 | 109 | ITGB2 | 50 | ETS1 | 30 | ADORA2A | 18 | ZC3H12A | 10 |
| NFKB1 | 107 | LCN2 | 49 | NQO1 | 30 | CD79A | 18 | BACH2 | 9 |
| EGFR | 106 | TLR7 | 49 | BMP2 | 29 | SERPINA3 | 18 | FDXR | 9 |
| MMP9 | 106 | CXCL2 | 48 | HSPA1B | 29 | TF | 18 | HSPA6 | 9 |
| TGFB1 | 102 | SQSTM1 | 48 | IRF5 | 29 | TNFRSF10A | 18 | LGALS1 | 9 |
| IL1A | 100 | EGR1 | 47 | NTRK2 | 29 | CYP27B1 | 17 | PFN1 | 9 |
| ICAM1 | 97 | C3 | 46 | BCL2A1 | 28 | RXRA | 17 | EPHX2 | 8 |
| CD44 | 87 | SOD2 | 46 | CXCL16 | 28 | MT-CO2 | 16 | KCNQ1 | 8 |
| ITGAM | 82 | APOB | 45 | TNFRSF10B | 28 | PLA2G4A | 16 | OPTN | 8 |
| NFKBIA | 82 | ENG | 45 | ADRB2 | 27 | CYP19A1 | 15 | TNIP1 | 8 |
| VCAM1 | 81 | FLT1 | 45 | CYP1A1 | 27 | CYP2C8 | 15 | ABCC3 | 7 |
| CCL5 | 79 | PLAU | 45 | CYP2E1 | 27 | DUOX1 | 15 | ASS1 | 7 |
| CXCL10 | 79 | SERPINA1 | 45 | GDF15 | 27 | HBB | 15 | HRH1 | 7 |
| TLR2 | 78 | VTN | 44 | PTGS1 | 27 | MSN | 15 | MAP2K6 | 7 |
| CSF2 | 77 | HBEGF | 43 | PTX3 | 27 | SGK1 | 15 | SLC18A2 | 7 |
| AGT | 76 | NOTCH1 | 43 | NPPA | 26 | CASP4 | 14 | FMO3 | 6 |
| CXCL12 | 74 | A2M | 42 | PPP1R15A | 26 | FABP1 | 14 | GLS2 | 6 |
| SERPINE1 | 73 | IL6R | 42 | OLR1 | 25 | FTH1 | 14 | KCNJ2 | 6 |
| CASP1 | 72 | NOD2 | 42 | TREM2 | 25 | GAL | 14 | SESN2 | 6 |
| HMOX1 | 67 | SOX2 | 42 | COL18A1 | 24 | MT-ATP6 | 14 | SORL1 | 6 |
| TIMP1 | 67 | TNFRSF1B | 41 | GADD45A | 24 | MT-CYB | 14 | TFEB | 6 |
| CSF3 | 65 | VEGFC | 41 | JUNB | 24 | SFTPD | 14 | TYRP1 | 6 |
| CSF1 | 64 | CD38 | 40 | KLF2 | 24 | TRIM21 | 14 | DMD | 5 |
| TNFRSF1A | 61 | BCL6 | 39 | MME | 24 | BBC3 | 13 | DRD4 | 5 |
| CD274 | 59 | FAS | 39 | TLR6 | 24 | FTL | 13 | LCAT | 5 |
| IRF1 | 59 | IL33 | 39 | CP | 23 | GP1BA | 13 | FRZB | 4 |
| B2M | 57 | ATF3 | 38 | DDIT3 | 23 | MT-ND1 | 13 | PDE4A | 4 |
| EDN1 | 57 | KLF4 | 37 | HPX | 23 | PRNP | 13 | ADH1A | 3 |
| SELE | 57 | SOCS1 | 37 | NAMPT | 23 | GCLM | 12 | ADH1C | 3 |
| CEBPB | 56 | TGFA | 37 | NPY | 23 | NEFL | 12 | ENDOG | 3 |
| THBS1 | 56 | PLAT | 36 | BAX | 22 | SLC7A11 | 12 | PLD1 | 3 |
| CASP8 | 55 | LTF | 35 | CIITA | 22 | TPM1 | 12 | ABCC8 | 2 |
| IL15 | 55 | MMP14 | 35 | MAOB | 22 | VIP | 12 | ACOX2 | 2 |
| TLR3 | 55 | RUNX2 | 35 | RAC2 | 22 | ACO1 | 11 | OSGIN1 | 2 |
| KIT | 54 | AGTR1 | 34 | APOH | 21 | BAG3 | 11 | PDLIM4 | 2 |
| MMP3 | 54 | F2 | 34 | RBP4 | 21 | BDKRB2 | 11 | RYR3 | 2 |
| PLG | 54 | HLA-A | 34 | CYP2C9 | 20 | GAP43 | 11 | SCN4B | 2 |
| CCR7 | 53 | IL11 | 34 | GADD45B | 20 | MT-CO3 | 11 | JAZF1 | 1 |
| GFAP | 52 | HSPA8 | 33 | HLA-B | 20 | MT-ND4 | 11 | DUSP19 | 0 |
|  |  |  |  | KRT18 | 20 | TTN | 11 | TPK1 | 0 |

**Table S4 Primers for qRT-PCR**

| Gene | Primer | Sequence |
| --- | --- | --- |
| IL1B | Forward | 5'-AGCTACGAATCTCCGACCAC-3' |
|  | Reverse | 5'-CGTTATCCCATGTGTCGAAGAA-3' |
| CXCL8 | Forward | 5'-ATGACTTCCAAGCTGGCCGTGGCT-3' |
|  | Reverse | 5'-TCTCAGCCCTCTTCAAAAACTTCTC-3' |
| CCL2 | Forward | 5'-CAGCCAGATGCAATCAATGCC-3' |
|  | Reverse | 5'-TGGAATCCTGAACCCACTTCT-3' |
| IL1A | Forward | 5'-TGGTAGTAGCAACCAACGGGA-3' |
|  | Reverse | 5'-ACTTTGATTGAGGGCGTCATTC-3' |
| ICAM1 | Forward | 5'-ATGCCCAGACATCTGTGTCC-3' |
|  | Reverse | 5'-GGGGTCTCTATGCCCAACAA-3' |
| CXCL10 | Forward | 5'-GTGGCATTCAAGGAGTACCTC-3' |
|  | Reverse | 5'-TGATGGCCTTCGATTCTGGATT-3' |
| NFKB1 | Forward | 5'-GGTGCGGCTCATGTTTACAG-3' |
|  | Reverse | 5'-GATGGCGTCTGATACCACGG-3' |
| TLR2 | Forward | 5'-CCTCTCGGTGTCGGAATGTC-3' |
|  | Reverse | 5'-TCCCGCTCACTGTAAGAAACA-3' |
| CCL5 | Forward | 5'-CCAGCAGTCGTCTTTGTCAC-3' |
|  | Reverse | 5'-CTCTGGGTTGGCACACACTT-3' |
| CCR7 | Forward | 5'-TGAGGTCACGGACGATTACAT-3' |
|  | Reverse | 5'-GTAGGCCCACGAAACAAATGAT-3' |
| GAPDH | Forward | 5'-GATTCCACCCATGGCAAATTC-3' |
|  | Reverse | 5'-CTGGAAGATGGTGATGGGATT-3' |

**Transcriptional Profiling**

***Experimental method*** This experiment was performed by GENESWAN BIOTECHNOLOGY, china. The SH-SY5Y cell line was cultured routinely until it reached approximately 70-80% confluence in the culture dish. The culture medium was removed, and PBS, LPS (1ug/mL), along with UC@MOF@RB+MEM, were added to the cells. The cells were then illuminated for 30 min and incubated for an additional 12 h. After incubation, PBS was removed, and the cells were harvested for transcriptomic analysis, qRT-PCR, enzyme activity assay, and MDA measurement. Workflow for transcriptome was performed by GENESWAN BIOTECHNOLOGY, China. The total RNA was isolated using the FastPure Complex Tissue/Cell Total RNA Isolation Kit (Vazyme Biotech Co., Ltd). The quality and integrity of the RNA were assessed using a NanoDrop spectrophotometer (HIPIE). Library construction was conducted utilizing the Hieff NGS Ultima dual-mode mRNA Library Prep Kit. Qualified sequencing libraries were gradient diluted and mixed in accordance with the required sequencing volume. Then, they were denatured into single strands with NaOH and sequenced on the DNBSEQ-T7 system (MGI Tech, China).

***Transcriptome analysis flow*** 1. **Quality control** Samples were sequenced on the platform to get image files, which were transformed by the software of the sequencing platform into original data in FASTQ format (Raw Data). The adapter sequences, low-quality reads and undetermined bases were filtered out using software fastp [1], yielding high-quality sequences (Clean Data) for further analysis. 2. **Reads mapping** The filtered reads were mapped to the reference genome (hg38) using STAR [2]. The alignment results underwent evaluation and quality control through software RNA-SeQC [3]. 3. **Expression analysis** We employed StringTie2 [4,5] for the quantifying expression level of genes and standardized these levels using FPKM. 4. **Differential expression analysis** The difference expression of genes was analyzed by DESeq (v1.38.3) with screened conditions as follows: expression difference multiple |log2FoldChange| ≥ 1, adjusted P value < 0.05. 5. **Enrichment analysis** We mapped all the genes to Terms in the Gene Ontology database and calculated the numbers of differentially enriched genes in each Term. Using ClusterProfiler package to perform GO enrichment analysis on the differential genes (up DEGs / down DEGs), calculate P-value by hypergeometric distribution method (the standard of significant enrichment is P-value < 0.05, and find the GO term with significantly enriched differential genes to determine the main biological functions performed by differential genes. ClusterProfiler package in R (4.4.0) was used to carry out the enrichment analysis of the KEGG pathway of differential genes, focusing on the significant enrichment pathway with P-value < 0.05. 6. **Analysis of genes associated with inflammatory responses** We intersected 200 inflammation-related genes from GSEA with differentially expressed genes (DEGs), identifying those altered by Zn^2+^. The STRING database (https://string-db.org/) was utilized for protein interaction analysis to identify the hub genes. 7. **Analysis of genes associated with oxidative stress** We intersected 1398 oxidative stress-related genes from GeneCards (relevance score≥7) with DEGs to identify those altered by Zn^2+^. The STRING database (https://string-db.org/) was used for protein interaction analysis to pinpoint the hub genes.

**Reference**

1. Chen S, Zhou Y, Chen Y, Gu J. fastp: an ultra-fast all-in-one FASTQ preprocessor. *Bioinformatics*. 2018;34(17):i884-i90.

2. Dobin A, Davis CA, Schlesinger F, Drenkow J, Zaleski C, Jha S, Batut P, Chaisson M, Gingeras TR. STAR: ultrafast universal RNA-seq aligner. *Bioinformatics*. 2013;29(1):15-21.

3. DeLuca DS, Levin JZ, Sivachenko A, Fennell T, Nazaire MD, Williams C, Reich M, Winckler W, Getz G. RNA-SeQC: RNA-seq metrics for quality control and process optimization. *Bioinformatics*. 2012;28(11):1530-2.

4. Pertea M, Pertea GM, Antonescu CM, Chang TC, Mendell JT, Salzberg SL. StringTie enables improved reconstruction of a transcriptome from RNA-seq reads. *Nat Biotechnol*. 2015;33(3):290-5.

5. Kovaka S, Zimin AV, Pertea GM, Razaghi R, Salzberg SL, Pertea M. Transcriptome assembly from long-read RNA-seq alignments with StringTie2. *Genome Biol*. 2019;20(1):278.
